# A systematic review and meta-analysis of the effectiveness and safety of COVID-19 vaccination in older adults

**DOI:** 10.3389/fimmu.2023.1113156

**Published:** 2023-03-03

**Authors:** Kun Xu, Zihan Wang, Maorong Qin, Yangyu Gao, Na Luo, Wanting Xie, Yihan Zou, Jie Wang, Xingming Ma

**Affiliations:** ^1^ School of Health Management, Xihua University, Chengdu, China; ^2^ Faculty of Geosciences and Environmental Engineering, Southwest Jiaotong University, Chengdu, China; ^3^ School of Food and Biological Engineering, Xihua University, Chengdu, China; ^4^ Health Promotion Center, Xihua University, Chengdu, China

**Keywords:** COVID-19 vaccine, older adults, SARS-CoV-2, effectiveness, safety, meta-analysis

## Abstract

In the coronavirus disease 2019 (COVID-19) pandemic, vaccinations were essential in preventing COVID-19 infections and related mortality in older adults. The objectives of this study were to evaluate the effectiveness and safety of the COVID-19 vaccines in older adults. We systematically searched the electronic bibliographic databases of PubMed, Web of Science, Embase, Cochrane Library, ClinicalTrials.gov, Research Square, and OpenGrey, as well as other sources of gray literature, for studies published between January 1, 2020, and October 1, 2022. We retrieved 22 randomized controlled trials (RCTs), with a total of 3,404,696 older adults (aged over 60 years) participating, that were included in the meta-analysis. No significant publication bias was found. In the cumulative meta-analysis, we found that the COVID-19 vaccines were effective in preventing severe acute respiratory syndrome coronavirus 2 (SARS-CoV-2) infection (OR = 0.38, 95% CI = 0.23–0.65, *p* = 0.0004) and in reducing the number of COVID-19-related deaths (OR = 0.16, 95% CI = 0.10–0.25, *p* < 0.00001) in elderly people. Antibody seroconversion (AS) and geometric mean titer (GMT) levels significantly increased in vaccinated older adults [OR = 24.42, 95% CI = 19.29–30.92; standardized mean difference (SMD) = 0.92, 95% CI = 0.64–1.20, respectively]. However, local and systemic adverse events after COVID-19 vaccine administration were found in older adults (OR = 2.57, 95% CI = 1.83–3.62, *p* < 0.00001). Although vaccination might induce certain adverse reactions in the elderly population, the available evidence showed that the COVID-19 vaccines are effective and tolerated, as shown by the decrease in COVID-19-related deaths in older adults. It needs to be made abundantly clear to elderly people that the advantages of vaccination far outweigh any potential risks. Therefore, COVID-19 vaccination should be considered as the recommended strategy for the control of this disease by preventing SARS-CoV-2 infection and related deaths in older adults. More RCTs are needed to increase the certainty of the evidence and to verify our conclusions.

**Systematic review registration:**

https://www.crd.york.ac.uk/prospero/display_record.php?ID=CRD42022319698, identifier CRD42022319698.

## Introduction

The emergence and spread of coronavirus disease 2019 (COVID-19) brought about negative effects and unprecedented challenges that affected the physical and mental well-being of people worldwide (1). According to a World Health Organization (WHO) report, as of October 1, 2022, there have been more than 616.95 million cumulative cases of COVID-19 globally, including more than 6.5 million deaths (2). A meta-analysis showed that mortality increases from 9.5% in patients 60–69 years old up to 29.6% in those aged >80 years (3). In another study, adults aged 65 years and older were found to be 8.7 times more likely to require hospitalization for severe acute respiratory syndrome coronavirus 2 (SARS-CoV-2) infection, and have accounted for 22% of cases and up to 78% of COVID-19-related deaths (4). Due to the lower efficacy of treatment for severe COVID-19, older adults have poorer clinical outcomes, including a greater chance of 30-day hospitalization and mechanical ventilation, which also potentially leads to higher mortality rates (5).

As a result, there was a critical need to focus on the vaccination of older adults against SARS-CoV-2 infection and to lower their risk of severe disease and mortality. Although various vaccines and treatments have continuously emerged, these have remained unable to completely control the spread of the virus and eliminate infections. After COVID-19 infection, bodily injury becomes very serious, especially in elderly people (6–11). In the absence of definitive treatment, the development of vaccines against COVID-19 was perceived as an effective strategy to contain the spread of the pandemic.

As of October 1, 2022, there were 177 COVID-19 vaccines in clinical development, 199 in preclinical development, and 11 in phase 4 clinical trials (12). At this time, the WHO had approved 11 COVID-19 vaccines for emergency use listing (EUL), which are shown in **Appendix 1** (13). More and more vaccines are now being approved for marketing and undergoing evaluation by the WHO EUL/PQ. The status of each of the 44 COVID-19 vaccines within the WHO EUL/PQ evaluation process is shown in **Appendix 2** (14).

COVID-19 vaccines have become more readily available all around the world. Timely vaccination and high vaccination rates are necessary to effectively control diseases (15). Thus far, a total of 163.2 billion vaccine doses have been administered and 28 people per 100 population boosted worldwide, with China having the highest cumulative number of vaccine doses, followed by the United States (2).

Age is an important factor affecting the spread of and infection with COVID-19. Compared with young people, elderly people are more likely to be infected with COVID-19 and more likely to experience serious illness after infection, and their hospitalization rates and mortality rates after infection are higher than those of young people (8–10). Since older adults have the highest rates of COVID-19 mortality, many countries have invested more resources into finding better strategies to achieve and sustain higher vaccination coverage in the older adult population (16). However, there is still disbelief and hesitation about the effectiveness of COVID-19 vaccines. Vaccine hesitancy and rejection (VHR) is one of the top 10 threats to global health (17, 18). The effectiveness and safety of the COVID-19 vaccines against COVID-19 infection therefore need to be assessed in older people.

Clinical trials have shown COVID-19 vaccines to be immunogenic against SARS-CoV-2 infection and safe, with their efficacy ranging from 86% and 95% for the messenger RNA (mRNA) vaccines BNT162b2 (19) and mRNA-1273 (20), respectively, to 74% for the AZD1222 (ChAdOx1 nCoV-19) vaccine (21) in those aged 18 years and older. The other vaccines included in this review have been found to have intermediate efficacies of 67% for Ad26.COV2.S (22) and 78% for the inactivated SARS-CoV-2 vaccine (23) in adults (aged ≥18 years). Furthermore, the COVID-19 vaccines have also been shown to be immunogenic against SARS-CoV-2 infection and safe in older adults (aged ≥60 years), a result similar to that seen in young and middle-aged people (aged from 18 to 60 years) (19–23). In some randomized controlled trials (RCTs), all vaccine formulations have been well tolerated overall, and vaccine-related adverse events or outcomes after vaccination have been generally mild to moderate and transient in adults (19–24).

Although the current COVID-19 vaccination guidelines for older people differ by country, the WHO recommends vaccination with the mRNA, recombinant adenovirus vector, or inactivated coronavirus vaccines, among others, for all older people (25). Older people, as a specific population, are at very high risk of adverse outcomes from infectious diseases due to comorbidities associated with aging and their decreased immunological competence (immunosenescence) (26). Immunosenescence not only increases susceptibility to SARS-CoV-2 infection but also limits the effectiveness of the COVID-19 vaccines, which may lead to differences in vaccine effectiveness between younger (<55 years old) and older people. Vaccine formulations effective in younger people might not engender immunity in older populations (27). Furthermore, there have been concerns that the currently available COVID-19 vaccines may not be adequate to protect older people from COVID-19 infection. A number of studies have revealed that older people had a higher rate of vaccination hesitancy and distrust compared to the general population owing to uncertainties and fears associated with vaccine side effects (28–30). Consequently, it is vital to conduct a meta-analysis on the efficacy and safety of the COVID-19 vaccines in older people, which would provide additional scientific data that could be helpful in protecting older people, a vulnerable demographic during the COVID-19 pandemic.

Therefore, in this meta-analysis and systematic review, we aimed to summarize the overall effectiveness and safety of the COVID-19 vaccines against COVID-19 infection in older people in order to provide evidence for an improved vaccine strategy for this population.

## Methods

### Data sources and search strategy

PubMed, Web of Science, Embase, Cochrane Library, ClinicalTrials.gov, Research Square, and open gray and gray literature were searched in the Chinese and English languages from January 1, 2020, to October 1, 2022. In addition, we also manually searched for articles that met the criteria. The search mesh terms included (“older adults” OR “old people” OR “old population” OR “the aged” OR “elder people” OR “the elderly” OR “older patients” OR “aging” OR “gerontology”) AND (“COVID-19” OR “coronavirus” OR “SARS-CoV-2” OR “variant strain” OR “Delta variant” OR “B.1.617.2” OR “Omicron variant” OR “B.1.1.529”) AND (“vaccine” OR “vaccination”) AND (“randomized controlled trial” OR “controlled clinical trial” OR “randomized” OR “randomly” OR “trial”), as shown in **Appendix 3**. Zotero 6.0.4 (https://www.zotero.org/) was used to manage and screen records and to exclude duplicates. This meta-analysis and systematic review were performed in strict accordance with the Preferred Reporting Items for Systematic Reviews and Meta-Analyses (PRISMA; checklist provided in **Appendix 4**). This study was registered on PROSPERO (CRD42022319698).

### Data selection criteria

This study included RCTs that evaluated the effectiveness and safety of COVID-19 vaccines in older adults (aged ≥60 years). Studies that met the following criteria were excluded: a) irrelevant to the subject of the meta-analysis (SARS-CoV-2 vaccination not involved); b) reviews, editorials, conference papers, case reports, or animal experiments; c) duplicate studies or studies with overlapping participants; d) unable to confirm diagnosis of COVID-19; and e) those with insufficient data to calculate the outcomes in terms of the effectiveness and safety of SARS-CoV-2 vaccines.

### Data screening and extraction

The references of the retrieved studies were screened to further select relevant studies suitable for inclusion in this meta-analysis. Data extraction was performed by two independent investigators based on the inclusion and exclusion criteria. The collected documents were processed as references using the document management software Zotero. Any disagreements were discussed with a third investigator. The following materials were extracted from each article by two independent investigators: a) basic information on the studies, including the first author, publication year, and study design; b) characteristics of the study population, including sample sizes, age groups, gender groups, and setting or locations; c) types of SARS-CoV-2 vaccines and the number of doses administered; d) outcomes in terms of the effectiveness of the SARS-CoV-2 vaccines, including the following: number of laboratory-confirmed COVID-19 infections, hospitalizations for COVID-19, admissions to the intensive care unit (ICU) for COVID-19, COVID-19-related deaths, number and sample origin of antibody titer or seroconversion rates, and number of interferon gamma (IFN-γ)-positive T cells; and e) outcomes in terms of the safety of the SARS-CoV-2 vaccines.

### Risk of bias and quality assessment

The Cochrane collaboration risk-of-bias tool RoB 2 IRPG beta v9 was used to assess all potential sources of bias in the included references (31), while the GRADE system was used to assess the quality of evidence for all systematic reviews (32, 33).

The Cochrane evaluation criteria include the following five aspects (31): randomization process, deviation from the intended intervention, missing outcome data, measurement of the outcome, and selection of reported results. Publication bias was visualized using funnel plots. Two reviewers (KX and ZW) independently assessed the risk of bias during the evaluation process. Any disagreements were resolved by negotiation or with the participation of a third reviewer (XM or JW). In accordance with the Cochrane Statement of Risk of Bias, the risk of bias for each study was rated as high, some concern, or low risk. Studies with a high overall risk of bias for any single outcome were excluded from the meta-analysis.

The quality of evidence according to the GRADE system was evaluated based on the following five aspects (32, 33): study design limitations, consistency between studies, directness (ability to generalize), precision of results (sufficient or precise data), and publication bias. In accordance with the scoring criteria of the GRADE system, the quality of evidence was classified into five levels: high, moderate, low, very low quality, or no evidence. Similarly, the GRADE system was used to classify the strength of recommendations into four levels: strong recommendation, weak recommendation, recommendation to use interventions only in research, or no recommendation (32, 33). Two reviewers (KX and ZW) independently assessed the studies on the GRADE system during the evaluation process. Any disagreements were resolved by negotiation or with the participation of a third reviewer (XM or JW). Studies with no evidence for any outcome were excluded from the meta-analysis.

### Outcomes

The outcomes were the evaluation of the effectiveness and safety of SARS-CoV-2 vaccines in older adults, which included vaccine effectiveness (VE), vaccine immunogenicity, and vaccine safety (VS). VE was defined as the percentage of participants infected by SARS-CoV-2 in relation to the total vaccinated population, with the infected group after vaccination including symptomatic individuals, laboratory-confirmed asymptomatic infections (infection after vaccination), individuals admitted to the hospital or ICU for COVID-19 (hospitalized or admitted to ICU after vaccination), and COVID-19-related deaths (death after vaccination). The immunogenicity of the vaccines was characterized by antibody seroconversion (AS) rate and geometric mean titer (GMT) of the relevant antibodies, which included neutralizing, anti-S (spike protein), and anti-RBD (spike protein receptor-binding domain) antibodies. VS was defined as the incidence of adverse events after the last vaccine dose had been administered, including total adverse events (AEs); solicited local adverse events (slAEs) such as pain, swelling, and redness; solicited systemic adverse events (ssAEs) such as fever, fatigue, and headache; and geriatric complications after vaccination.

### Statistical analysis

Statistical analysis was performed using the Cochrane collaboration review management software (RevMan5.4). Binary variables representing the effectiveness and safety of the SARS-CoV-2 vaccines in comparison with a control group were expressed as odds ratios (ORs) and 95% confidence intervals (CIs), while continuous variables for the same measures in comparison with a control group were expressed in the form of standardized mean differences (SMDs) and 95% CIs. Heterogeneity was identified using the inconsistency (*I* ^2^) metric. Degrees of statistical heterogeneity were considered to be low (*I* ^2^ < 30%), moderate (*I* ^2^ = 30%–50%), or high (*I* ^2^ > 50%). The possible sources of heterogeneity were explored using sensitivity analysis. In cases where *I* ^2^ was <50%, which represents low-to-moderate heterogeneity and no statistical heterogeneity among the studies, a fixed effects model was used. Otherwise, a random effects model was used for analysis (*I* ^2^ ≥ 50%, which represents statistical heterogeneity among the studies). Publication bias was examined using Egger’s regression test and a funnel plot visual test; this was measured only when a subgroup contained three or more studies. Values of *p* < 0.05 were considered to represent statistical significance.

## Results

### Systematic literature search

**Figure 1:**
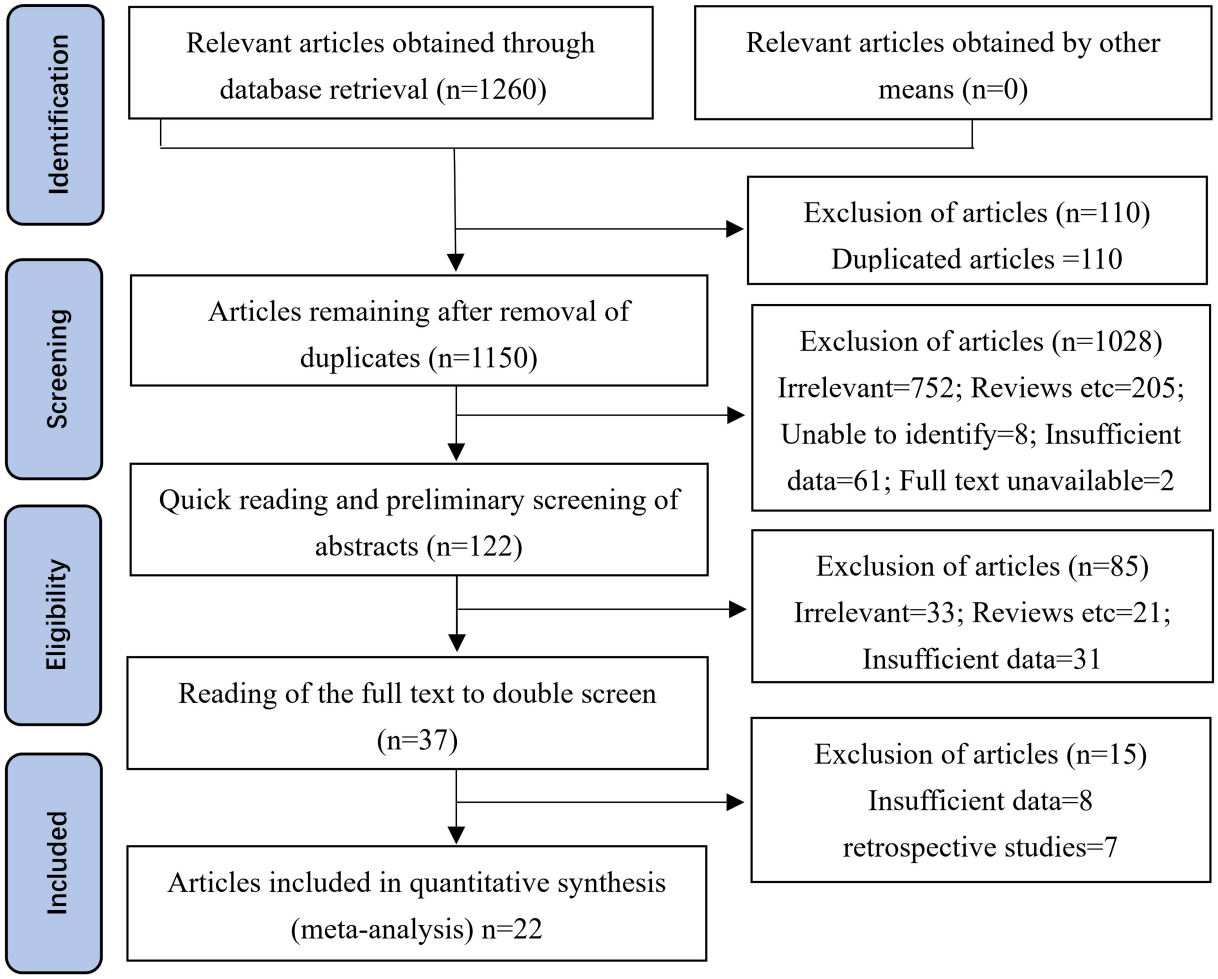
Methodological PRISMA (Preferred Reporting Items for Systematic Reviews and Meta-Analyses) flowchart for the selection of the studies included in this meta-analysis. A total of 306 studies were obtained from PubMed, 107 from Embase, 77 from the Cochrane Library, 100 from the Web of Science, 13 from ClinicalTrials.gov, 657 from Research Square, and 0 from OpenGrey or other sources of gray literature.

The PRISMA literature retrieval flowchart is shown in **Figure 1**.** **A total of 1,260 potentially relevant articles were identified up to October 1, 2022, from electronic databases, including 306 from PubMed, 107 from Embase, 77 from the Cochrane Library, 100 from Web of Science, 13 from ClinicalTrials.gov, 657 from Research Square, and 0 from OpenGrey or other sources of gray literature. After preliminary screening, 110 duplicate records were excluded. After reading the titles and abstracts, 1,028 publications were then excluded in accordance with the inclusion and exclusion criteria. Subsequently, after reading the abstract and full text of each publication in detail, another 100 records were excluded due to insufficient data, unavailability of the full text, or no confirmed diagnosis. Ultimately, 22 studies were included in this meta-analysis based on the inclusion criteria.

### Basic characteristics

A total of 22 articles were included in the meta-analysis (21, 34–54), as shown in **Table 1**. From these publications, the relevant indicators were extracted, including information on the author, year of publication, number of participants, and vaccination efficacy and safety. Four articles reported data related to vaccination effectiveness (21, 35, 37, 54), 16 articles reported data related to antibody titer levels after vaccination (34, 36, 38–46, 48–51, 53), and 13 articles reported data related to the occurrence of vaccine-related adverse events in elderly people (21, 36, 38, 39, 41, 43, 45–49, 51, 52).

**Table 1:** Basic features of the studies included in the meta-analysis.

Study	Study design	Vaccine name	Vaccine type	Number of doses	Research quantum (V/C)	Age (years)	Gender (M/F)	Outcome measures	RoB2
Poh et al. (34)	RCT	BNT162b2, mRNA-1273	NAV	Three doses	24/23 (BBB/BBM)	>60	22/25	GMT, GMTAT, GMTVT	L
Ioannou et al. (35)	RCT	BNT162b2, mRNA-1273, JNJ-78436735	VVV, NAV	Two doses	1,472,010/1,472,010	≥65	1,270,345/201,665	VE, VEVT, VEND	S
Formica et al. (36)	RCT	NVX-CoV2373	SV	Two doses	233/116	60-84	169/180	GMT, GMTAT, GMTVT, VS, slAE, ssAE	L
Sadoff et al. (37)	RCT	Ad26.COV2.S	VVV	One dose	6,403/6,340	≥60	NA	VE, VEVT, VEND	L
Lanini et al. (38)	RCT	GRAd-COV2	VVV	Two doses	45	65–85	NA	GMT, GMTAT, GMTVT, ssAE	S
Shu et al. (39)	RCT	V-01	SV	Two doses	360/80	60–83	262/178	AS, GMT, GMTAT, GMTVT, VS, slAE, ssAE	L
Alidjinou et al. (40)	RCT	BNT162b2	NAV	Two doses	47	77–90	17/30	GMT, GMTAT, GMTVT	L
Sadoff et al. (41)	RCT	Ad26.COV2.S	VVV	Two doses	320/88	65–88	190/211	GMT, GMTAT, GMTVT, VS, slAE, ssAE	L
Falsey et al. (21)	RCT	AZD1222	VVV	Two doses	4827/2411	≥65	NA	VE, VEVT, VEND, VS, slAE, ssAE	L
Zakarya et al. (42)	RCT	QazCOVID-in vaccine	IV	Two doses	100	≥60	35/65	AS	L
Guo et al. (43)	RCT	Sinopharm	IV	Three doses	252/84	≥60	200/136	AS, VS, slAE, ssAE	L
Ramasamy et al. (44)	RCT	ChAdOx1 nCoV-19	VVV	Two doses	156/40	≥70	103/74	GMT, GMTAT, GMTVT	L
Richmond et al. (45)	RCT	SCB-2019	SV	Two doses	73/18	55–75	36/55	AS, VS, slAE, ssAE	L
Li et al. (46)	RCT	BNT162b1	NAV	Two doses	48/24	65–85	36/36	AS, VS, slAE, ssAE	S
Walsh et al. (47)	RCT	BNT162b1	NAV	Two doses	72/18	65–82	30/60	slAE, ssAE	L
Wu et al. (48)	RCT	CoronaVac	IV	Two doses	347/74	≥70	206/215	AS, GMT, GMTAT, GMTVT, VS, slAE,ssAE	L
Wynne et al. (49)	RCT	ReCOV	SV	Two doses	10/40	56-80	26/24	VS, slAE, ssAE	L
Sáez-Llorens et al. (50)	RCT	mRNA-LNP	NAV	Two doses	306	>60	164/142	AS, GMT, GMTAT, GMTVT, VS	L
Sadoff et al. (51)	RCT	Ad26.COV2.S	VVV	Two doses	24	≥65	NA	GMT, GMTAT, GMTVT	L
Tanishima et al. (52)	RCT	KD-414	IV	Three doses	90/14	≥65	53/51	VS, slAE, ssAE	L
Kundro et al. (53)	RCT	rAd26/BBIBP-CorV, rAd26/ChAdOx1, rAd26/rAd5	IV, VVV	Two doses	27/27/31	≥65	33/48	GMT, GMTAT, GMTVT	L
Song et al. (54)	RCT	BBIBP-CorV/CoronaVac	IV	Two doses	68/113	66.5–74	74/107	VE, VEVT, VEND	L

RCT, randomized controlled trial; IV, inactivated vaccine; SV, subunit vaccine; VVV, viral vector vaccine; NAV, nucleic acid vaccine; V/C, vaccine group/control or placebo control group; M/F, male/female; NA, not available; VE, vaccine effectiveness (infection, hospitalization or ICU admission, death after vaccination); VEND, vaccine effectiveness (by number of doses); VEVT, vaccine effectiveness (by vaccine type); AS, antibody seroconversion; GMT, geometric mean titer; GMTAT, geometric mean titer (by antibody type); GMTVT, geometric mean titer (by vaccine type); VS, vaccine safety; slAE, solicited local adverse event; ssAE, solicited systemic adverse event; H, high overall bias; L, low overall bias; S, overall bias rating of “some concern”.

In addition to these, the same indicators (including author, year of publication, number of participants, and vaccine efficacy and safety) could be extracted from seven retrospective studies (55–61), shown in **Appendix 5**. Four articles reported on VE (58–61), while three articles reported antibody titer levels after vaccination (55–57) in the elderly. Six articles reported the effectiveness and safety of two vaccine doses (56–61), while only one article reported the effectiveness and safety of three vaccine doses (55) in elderly people. Five articles reported the effectiveness and safety of the nucleic acid vaccine (56, 58–61), while three articles reported the effectiveness and safety of the inactivated vaccine (55, 57, 58) in elderly people. Although vaccination was found to provide clear protection against SARS-CoV-2 infection in older adults, the results showed high and inexplicable heterogeneity in terms of both its effectiveness and its safety. Analyses of the data of these retrospective studies are presented in **Appendix 5**.

Finally, the same indicators (including author, year of publication, number of participants, and vaccination efficacy and safety) could be extracted from eight qualitative analysis articles (62–69), which are shown in **Appendix 6**. However, these studies could not be included in the meta-analysis due to insufficient data and/or descriptive explanations of vaccination efficacy and safety without the use of a parallel control, among other reasons. One article reported the VE (63), three articles reported antibody titer levels after vaccination (64, 65, 69), and six articles reported the occurrence of vaccine-related adverse events in the elderly (62, 64–68).

### Quality assessment

**Figure 2:**
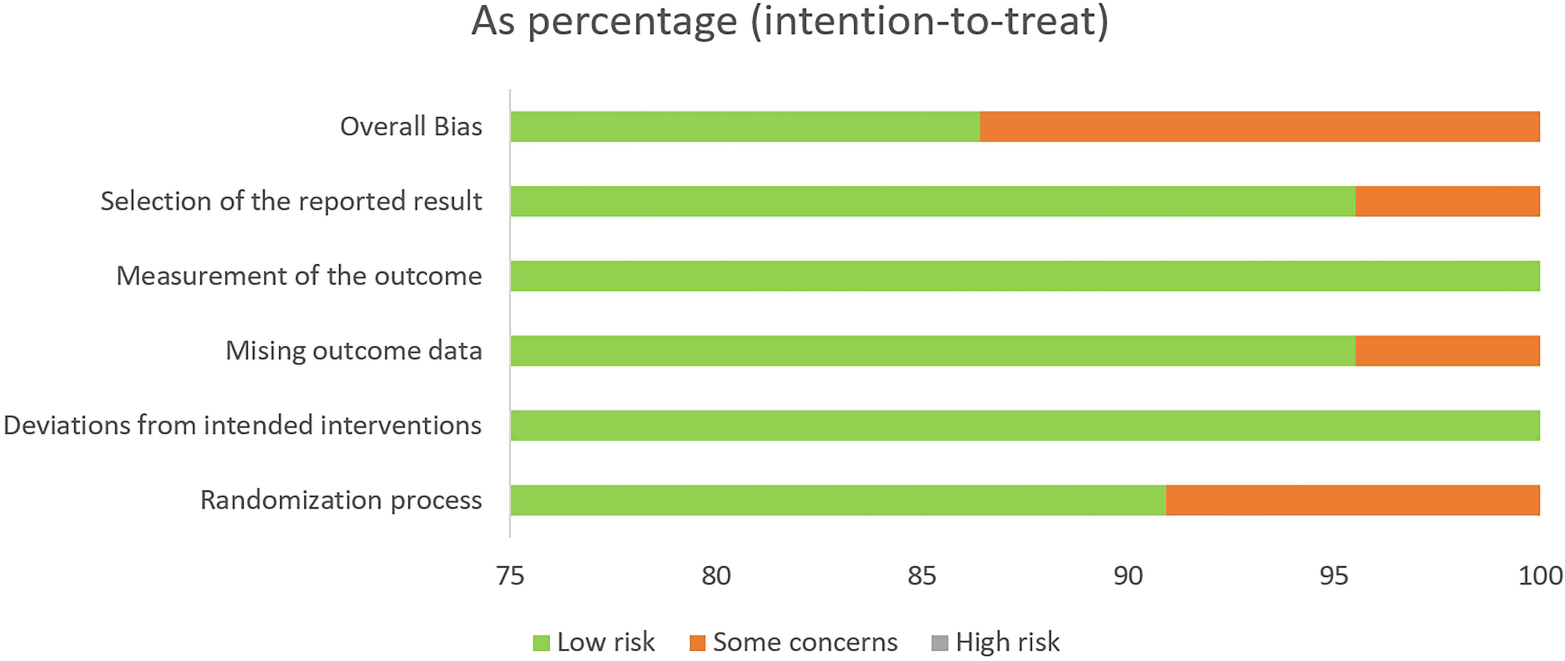
Risk of bias in the randomized controlled trials (RCTs) included, assessed using the Cochrane RoB2 tool.

The Cochrane Risk-of-Bias 2 tool (RoB 2 v9) was used to evaluate the quality of the individual studies included (**Appendix 7**). After evaluation, 19 articles were rated as low risk (21, 34, 36, 37, 39–45, 47–54), while the risk of bias for three articles was rated as “some concern” (35, 38, 46), as shown in **Table 1** and **Figure 2**. Additionally, we used GRADEprofiler 3.6 to assess the quality of evidence for all systematic reviews. Of all the pieces of evidence included in the analysis, nine were characterized as high-quality evidence [VE, VEND (vaccine effectiveness by number of doses), VEVT (vaccine effectiveness by vaccine type), GMT, GMTAT (geometric mean titers by antibody type), GMTVT (geometric mean titers by vaccine type), AE, slAE, and ssAE], while one (AS) was characterized as evidence of moderate quality (**Appendix 8**). According to the quality evaluation using GRADE of the evidence on outcomes in terms of VE, immunogenicity, and VS, COVID-19 vaccination in older adults should be considered to be a strongly recommended strategy for control of COVID-19 through prevention of SARS-CoV-2 infection and reduction of COVID-19-related deaths.

### Heterogeneity and risk of bias

**Figure 3:**
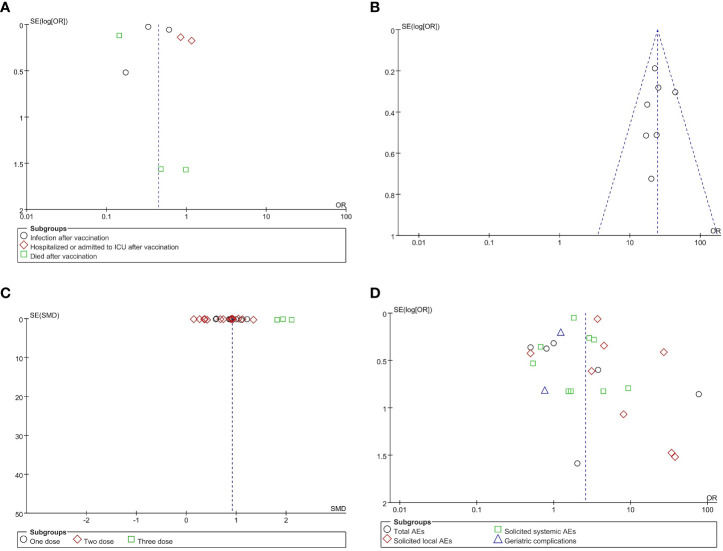
Funnel plots for publication bias. Publication bias in reports of vaccine effectiveness (VE) **(A)**, antibody seroconversion (AS) **(B)**, geometric mean titer (GMT) **(C)**, and vaccine safety (VS) **(D)**. *OR*, odds ratio; *SMD*, standardized mean difference.

Prior to meta-analysis of the included articles, a heterogeneity test was performed for results in which there were two or more included papers. The results of the analysis showed that no study significantly interfered with the results of the meta-analysis. The risk of publication bias was evaluated through funnel plots produced using Revman5.3; evidence of significant publication bias can be ignored due to the good levels of symmetry observed in these funnel plots. The shapes of the funnel plots for VE, AS, GMT, and VS are shown in **Figure 3**, while those of the subgroups are shown in **Appendix 9**. Groups with heterogeneity scores over 50 (*I* ^2^ > 50%) were examined using Egger’s test; the results showed no evidence of publication bias (*p* > 0.05), except in the cases of GMT, anti-S of GMTAT, and nucleic acid vaccine of GMTVT groups (*p* < 0.05). Data from Egger’s test are shown in **Appendix 10**.

### Effectiveness of COVID-19 vaccines among older adults

#### Vaccine effectiveness

**Figure 4:**
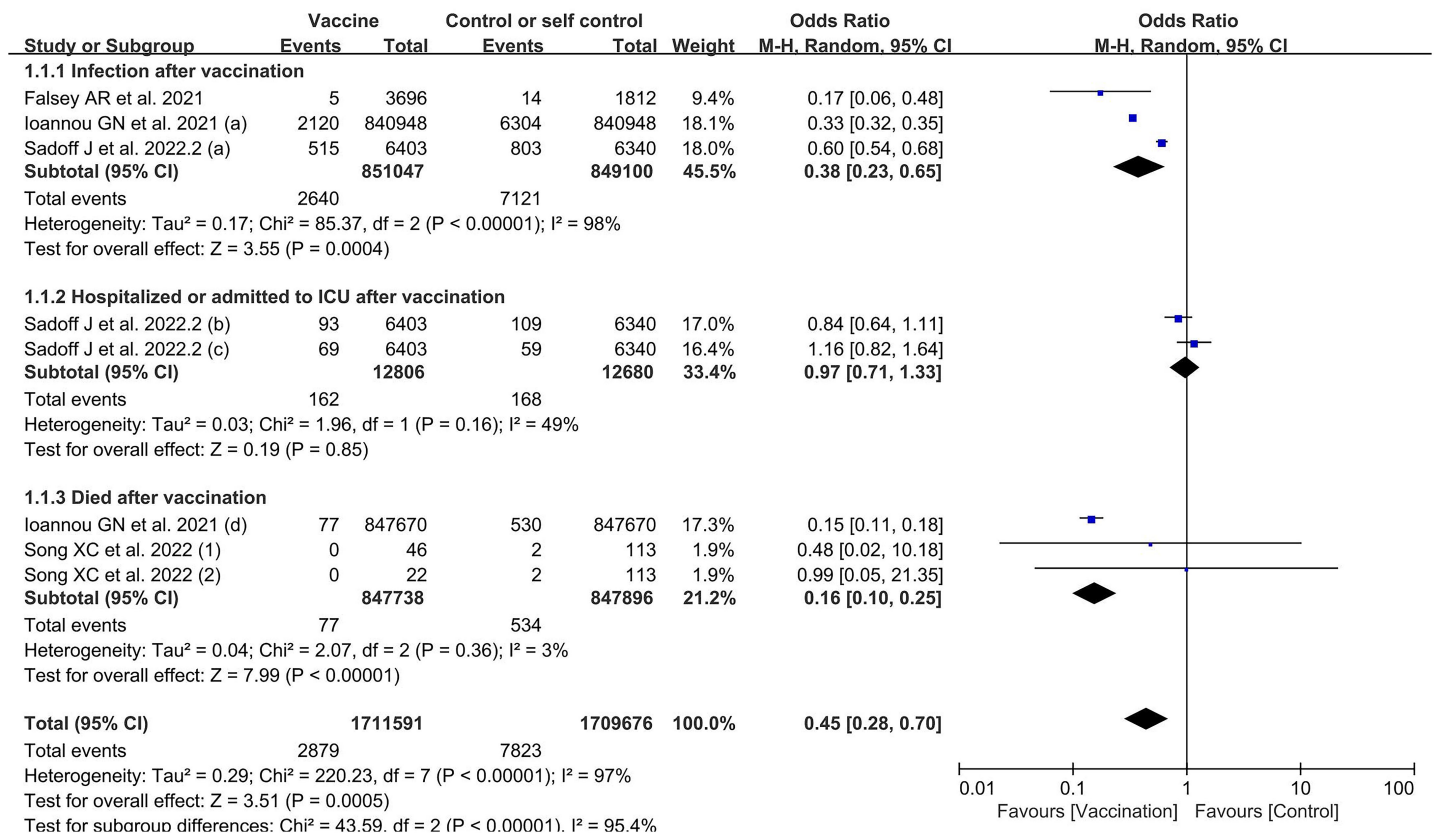
Forest plots of vaccine effectiveness by number of doses for the coronavirus disease 2019 (COVID-19) vaccine group compared with the control group. (a), infection after vaccination; (b), hospitalized after vaccination; (c), admitted to ICU after vaccination; (d), death after vaccination; *1*, one dose; *2*, two doses.

Four included studies contained data related to VE; these included a total of 1,711,591 and 1,709,676 participants in the vaccine and control groups, respectively. A random effects model was used for the meta-analysis due to the high heterogeneity (*p* < 0.00001, *I* ^2^ = 95.4%) of the data (**Figure 4**). The meta-analysis on the effectiveness of the vaccine in this group of studies indicated an OR representing lower risk in the vaccine group compared to the control group (OR = 0.45, 95% CI = 0.28–0.70, *p* = 0.0005). The COVID-19 vaccines were shown to be more effective in preventing SARS-CoV-2 infection (OR = 0.38, 95% CI = 0.23–0.65, *p* = 0.0004) and in reducing COVID-19-related deaths (OR = 0.16; 95% CI = 0.10–0.25, *p* < 0.00001), but less effective in preventing hospitalization and ICU treatment (OR = 0.97, 95% CI = 0.71–1.33, *p* = 0.85) in elderly people. The subgroup analysis for each effectiveness indicator is shown in **Appendix 11**.

#### Subgroup analysis for vaccine effectiveness

The subgroup analysis for number of doses identified significant differences among four studies in the effects observed in experiments using one and two doses, which revealed that two vaccination doses had better effectiveness against SARS-CoV-2 infection compared to only one vaccination dose (*χ* ^2^ = 10.24, *p* = 0.001, *I* ^2^ = 90.2%) (**Table 2** and **Appendix 11**). The outcomes demonstrated that the vaccinated group experienced better outcomes than the control group in both one-dose (OR = 0.81, 95% CI = 0.56–1.17, *p* = 0.26) and two-dose experiments (OR = 0.23, 95% CI = 0.11–0.45, *p* < 0.0001).

**Table 2:** Subgroup analysis for vaccine effectiveness.

Study characteristics	Data			Test for heterogeneity			Test for effect		Subgroup	
	No. of studies	Vaccine	Control	*I* ^2^ (%)	Chi-squared test	*p*-value	OR (CI)	*p*-value	Statistic	*p*-value
No. doses										
One dose	4	19,255	19,133	80	15.24	0.002	0.81 (0.56–1.17)	0.26	10.24	0.001
Two doses	4	1,692,336	1,690,543	94	47.05	<0.00001	0.23 (0.11–0.45)	<0.00001		
Total	8	1,711,591	1,709,676	97	220.23	<0.00001	0.45 (0.28–0.70)	0.0005		
Vaccine type										
IV	2	68	226	0	0.11	0.74	0.69 (0.08–6.00)	0.74	5.90	0.05
NAV	2	1,688,618	1,688,618	98	45.14	<0.00001	0.22 (0.10–0.50)	0.0003		
VVV	4	22,905	20,832	86	21.78	<0.0001	0.69 (0.46–1.05)	0.08		
Total	8	1,711,591	1,709,676	97	220.23	<0.00001	0.45 (0.28–0.70)	0.0005		

IV, inactivated vaccine; VVV, viral vector vaccine; NAV, nucleic acid vaccine.

The subgroup analysis for vaccine type identified significant differences among four studies in the effects observed in experiments on the inactivated, nucleic acid, and viral vector vaccine groups (**Table 2** and **Appendix 11**). The subgroup of studies with the nucleic acid vaccine showed better effectiveness compared to studies with the viral vector and inactivated vaccines (*χ* ^2^ = 5.90, *p* = 0.05, *I* ^2^ = 66.1%). The outcomes demonstrated that the vaccinated group experienced better outcomes than the control group in the experiments with inactivated (OR = 0.69, 95% CI = 0.08–6.00, *p* = 0.74), nucleic acid (OR = 0.22, 95% CI = 0.10–0.50, *p* = 0.0003), and viral vector vaccines (OR = 0.69, 95% CI = 0.46–1.05, *p* = 0.08).

### Immunogenicity of COVID-19 vaccines among older adults

#### Antibody seroconversion rate

**Figure 5:**
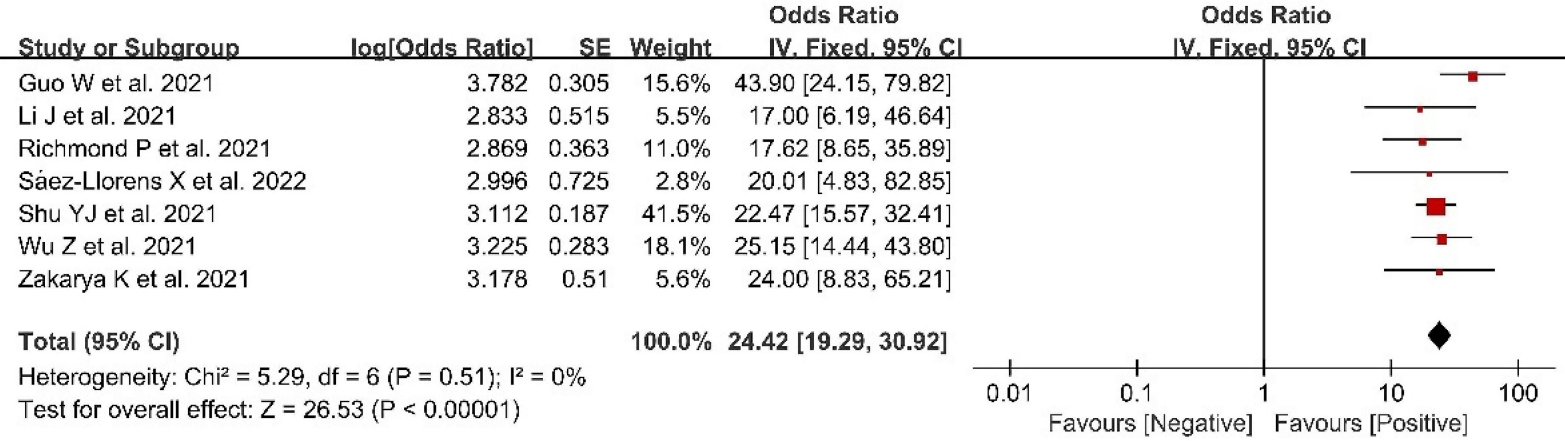
Forest plots of antibody seroconversion rate after coronavirus disease 2019 (COVID-19) vaccination.

Seven included studies presented data related to AS rate; these included 1,584 participants in vaccine groups (**Figure 5**). A fixed effects model was used for the meta-analysis due to the low heterogeneity (*p* = 0.51, *I* ^2^ = 0) of the data. The meta-analysis in the vaccine group found an OR indicating higher AS rates in the vaccinated groups compared with the control groups (OR = 24.42, 95%CI = 19.29–30.92, *p* < 0.00001).

#### Geometric mean titer

**Figure 6:**
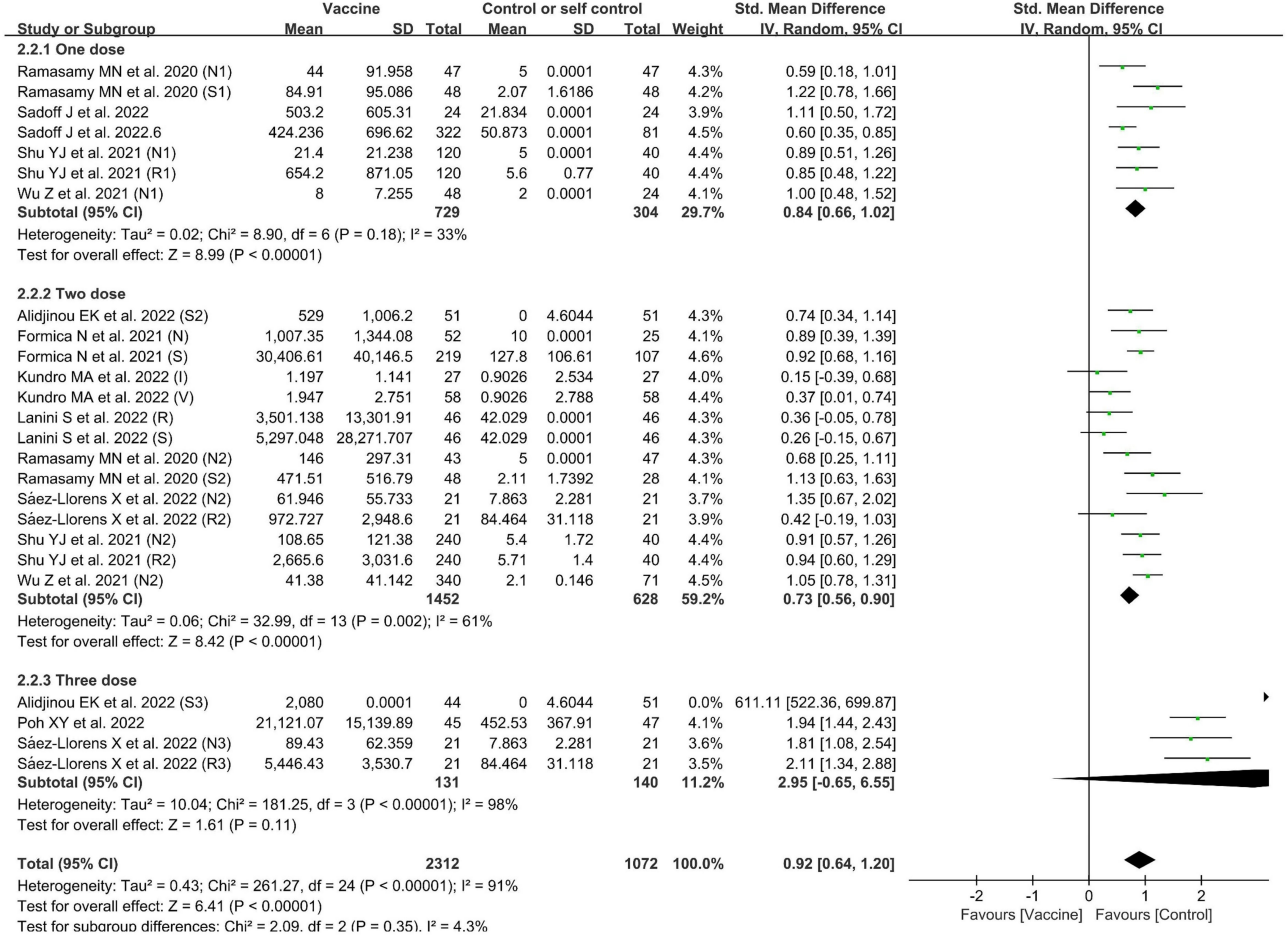
Forest plots of geometric mean titers (GMT) by number of doses for the coronavirus disease 2019 (COVID-19) vaccine group compared with the control group. *N*, neutralizing antibody; *S*, anti-S antibody; *R*, anti-RBD antibody; *1*, one dose; *2*, two doses; *3*, three doses; *I*, inactivated vaccine; *V*, viral vector vaccine.

There were 11 included studies with data related to GMT; these included 2,312 and 1,072 participants in the vaccine and control groups, respectively (**Figure 6**). A random effects model was used for the meta-analysis due to the significant level of statistical heterogeneity (*p* < 0.00001, *I* ^2^ = 91% > 50%) among studies. The pooled effects of these studies (SMD = 0.92, 95% CI = 0.64–1.20, *Z* = 6.41, *p* < 0.00001) showed that antibody titer levels improved significantly in the vaccine group, with a large effect compared to the control group. In addition, the subgroup analysis for number of doses found significant differences among 11 studies in the effects of experiments in which one dose, two doses, and three doses were administered (*χ* ^2^= 2.09, *p* = 0.35, *I* ^2^ = 4.3%). The three-dose subgroup showed better effectiveness than both the one-dose and two-dose subgroups. The outcomes demonstrated that the vaccine group experienced better outcomes than the control group in the experiments involving one dose (SMD = 0.84, 95% CI = 0.66–1.02, *Z* = 8.99, *p* < 0.00001), two doses (SMD = 0.73, 95% CI = 0.56–0.90, *Z* = 8.42, *p* < 0.00001), and three doses (SMD = 2.95, 95% CI = −0.65 to 6.55, *Z* = 1.61, *p* = 0.11).

#### Subgroup analysis for GMT

The subgroup analysis of GMT for different antibody types found no statistical differences among the subgroups of neutralizing, anti-S, and anti-RBD antibodies (*χ* ^2^= 0.32, *p* = 0.85, *I* ^2^ = 0) (**Table 3** and **Appendix 11**). The outcomes demonstrated that the vaccine group experienced better outcomes than the control group in results pertaining to neutralizing antibodies (SMD = 0.82, 95% CI = 0.64–1.01, *Z* = 8.73, *p* < 0.00001), anti-S antibodies (SMD = 1.11, 95% CI = 0.08–2.15, *Z* = 2.10, *p* = 0.004), and anti-RBD antibodies (SMD = 0.88, 95% CI = 0.44–1.31, *Z* = 3.94, *p* < 0.0001).

**Table 3:** Subgroup analysis for geometric mean titers (GMT).

Study characteristics	Data			Test for heterogeneity			Test for effect		Subgroup	
	No. of studies	Vaccine	Control	*I* ^2^ (%)	Chi-squared test	*p*-value	SMD (CI)	*p*-value	Statistic	*p*-value
Antibody type										
Neutralizing antibody	13	1,363	526	60	30.21	0.003	0.82 (0.64–1.01)	<0.00001	0.32	0.85
Anti-S	7	501	378	97	210.49	<0.00001	1.11 (0.08–2.15)	0.04		
Anti-RBD	5	448	168	77	17.65	0.001	0.88 (0.44–1.31)	<0.0001		
Total	25	2,312	1,072	91	261.27	<0.00001	0.92 (0.64–1.20)	<0.00001		
Vaccine type										
IV	3	415	122	78	8.92	0.01	0.76 (0.23–1.29)	0.005	4.28	0.23
SV	6	991	292	0	0.15	1.00	0.91 (0.77–1.04)	<0.00001		
NAV	7	224	233	97	209.12	<0.00001	1.57 (0.04–3.11)	0.04		
VVV	9	682	425	60	19.87	0.01	0.67 (0.46–0.88)	<0.00001		
Total	25	2,312	1,072	91	261.27	<0.00001	0.92 (0.64–1.20)	<0.00001		

Anti-S, anti-spike protein antibody; Anti-RBD, anti-RBD (spike protein receptor-binding domain) antibody; GMT, geometric mean titers; IV, inactivated vaccine; SV, subunit vaccine; VVV, viral vector vaccine; NAV, nucleic acid vaccine.

Although the nucleic acid vaccine showed better effectiveness compared to the inactivated, subunit, and viral vector vaccines (**Table 3** and **Appendix 11**), the subgroup analysis for vaccine type found no statistical differences among 11 studies in the effects of experiments involving subunit, nucleic acid, and viral vector vaccine subgroups (*χ* ^2^= 4.28, *p* = 0.23, *I* ^2^ = 29.9%). The outcomes demonstrated that the vaccine group experienced better outcomes than the control group in the case of experiments involving inactivated vaccines (SMD = 0.76, 95% CI = 0.23–1.29, *Z* = 2.82, *p* = 0.005), subunit vaccines (SMD = 0.91, 95% CI = 0.77–1.04, *Z* = 12.88, *p* < 0.00001), nucleic acid vaccines (SMD = 1.57, 95% CI = 0.04–3.11, *Z* = 2.01, *p* = 0.004), and viral vector vaccines (SMD = 0.67, 95% CI = 0.46–0.88, *Z* = 6.13, *p* < 0.00001).

### Safety of COVID-19 vaccines among older adults

#### Vaccine safety

**Figure 7:**
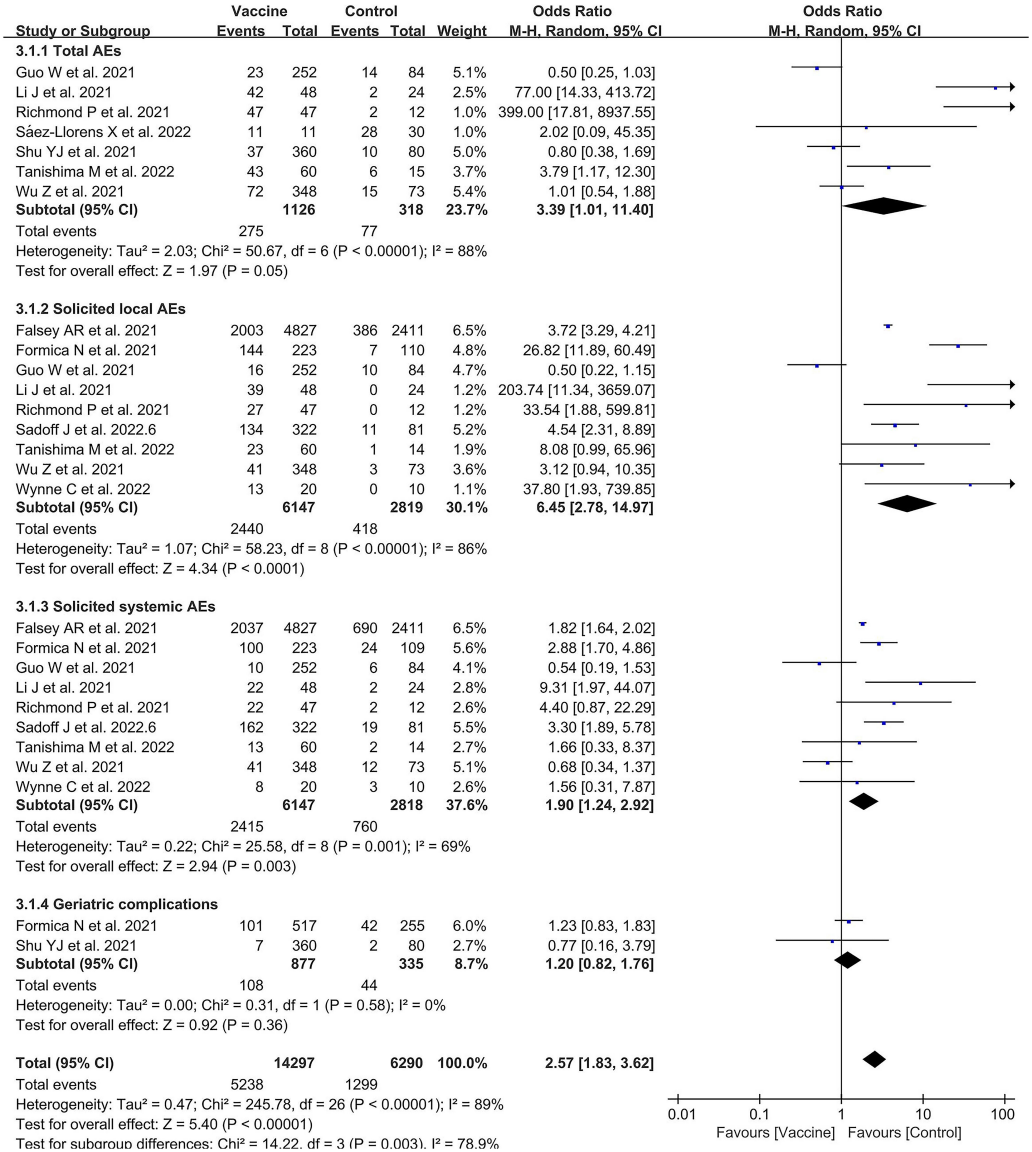
Forest plots of vaccine safety for the coronavirus disease 2019 (COVID-19) vaccine group compared with the control group.

There were 10 included studies with data related to vaccine-related adverse events; these included 14,297 and 6,290 participants in the vaccine and control groups, respectively (**Figure 7**). A random effects model was used for the meta-analysis due to the high heterogeneity (*p* < 0.00001, *I* ^2^ = 89%) of the data. The meta-analysis found an OR reflecting higher odds of adverse events in the vaccine group compared to the control group (OR = 2.57, 95% CI = 1.83–3.62, *p* < 0.00001). In addition, the subgroup analysis for immune effect found significant differences among 10 studies in the effects of experiments examining total AEs, slAEs, ssAEs, and geriatric complications after vaccination (*χ* ^2^= 14.22, *p* = 0.003, *I* ^2^ = 78.9%) (**Figure 7**). The outcomes demonstrated that the vaccine group experienced more AEs than the control group in the experiments on AEs (OR = 3.39, 95%CI = 1.01–11.40, *p* = 0.05), slAEs (OR = 6.45, 95%CI = 2.78–14.97, *p* < 0.0001), ssAEs (OR = 1.90, 95%CI = 1.24–2.92, *p* = 0.003), and geriatric complications (OR = 1.20; 95%CI = 0.82–1.76, *p* = 0.36).

#### Subgroup analysis for vaccine safety

A total of 10 studies included data related to the VS in terms of slAEs; these included 14,127 and 6,168 participants in the vaccine and control groups, respectively (**Table 4** and **Appendix 11**). The random effects model was used for the meta-analysis due to the higher heterogeneity (*p* < 0.00001, *I* ^2^ = 73%) of the data. The meta-analysis found an OR reflecting higher odds of slAEs in the vaccine group compared to the control group (OR = 3.82, 95% CI = 2.19–6.65, *p* < 0.00001). In addition, the subgroup analysis for immune effect found no statistical differences among the 10 studies in incidence of pain (OR = 5.04, 95% CI = 2.15–11.83, *p* = 0.0002), swelling (OR = 3.31, 95% CI = 0.89–12.28, *p* = 0.07), or redness (OR = 3.13, 95% CI = 0.90–10.94, *p* =0.07), *χ* ^2^= 0.51, *p* = 0.78, *I* ^2^ = 0.

**Table 4:** Subgroup analysis for vaccine safety.

Study characteristics	Data			Test for heterogeneity			Test for effect		Subgroup	
	No. of studies	Vaccine	Control	*I* ^2^ (%)	Chi-squared test	*p*-value	OR (CI)	*p*-value	Statistic	*p*-value
Solicited local AEs										
Pain	11	6,579	2,918	86	70.13	<0.00001	5.04 (2.15–11.83)	0.0002	0.51	0.78
Swelling	8	1,329	412	51	14.33	0.05	3.31 (0.89–12.28)	0.07		
Redness	10	6,219	2,838	61	22.89	0.006	3.13 (0.90–10.94)	0.07		
Total	29	14,127	6,168	73	101.89	<0.00001	3.82 (2.19–6.65)	<0.00001		
Solicited systemic AEs										
Fever	12	6,862	2,998	24	14.45	0.21	5.38 (2.79–10.37)	<0.00001	17.45	0.0002
Fatigue	10	6,327	2,833	42	15.40	0.08	1.65 (1.46–1.86)	<0.00001		
Headache	10	6,356	2,808	0	5.83	0.76	2.12 (1.85–2.44)	<0.00001		
Total	32	19,545	8,639	31	45.22	0.05	1.91 (1.75–2.09)	<0.00001		

AEs, adverse events.

There were 12 included studies with data related to the VS in terms of ssAEs; these included 19,545 and 8,639 participants in the vaccine and control groups, respectively (**Table 4** and **Appendix 11**). A fixed effects model was used for the meta-analysis due to the lower heterogeneity (*p* = 0.05, *I* ^2^ = 31%) of the data. The meta-analysis found a higher OR in the vaccine group compared to the control group (OR = 1.91, 95%CI = 1.75–2.09, *p* < 0.00001). In addition, the subgroup analysis for immune effect found differences among the 10 studies in terms of the effects on fever (OR = 5.38, 95% CI = 2.79–10.37, *p* < 0.00001), fatigue (OR = 1.65, 95% CI = 1.46–1.86, *p* < 0.00001), and headache (OR = 2.12, 95% CI = 1.85–2.44, *p* < 0.00001), *χ* ^2^= 17.45, *p* = 0.0002, *I* ^2^ = 88.5%.

## Discussion

In this study, we assessed the effectiveness of COVID-19 vaccines against COVID-19 infection and their safety among older people. In the analysis of VE, we found that any dose of the vaccine is protective in elderly people; however, administration of two doses is more effective than one dose. The nucleic acid vaccines are more effective than other types, inactivated and viral vector vaccines, with the inactivated vaccine being the least effective of these three types. These three types of COVID-19 vaccine were more effective in preventing SARS-CoV-2 infection and in reducing deaths after infection, but less effective in preventing hospitalization and ICU treatment. It was found that elderly people who received COVID-19 vaccines experienced better outcomes (or the same level of outcome) than those who did not receive a COVID-19 vaccine in all aspects. It can be considered that vaccination for COVID-19 is still the major strategy for the prevention of SARS-CoV-2 infection and reduction of the severity of illness after infection. In terms of immunogenicity, the meta-analysis revealed high AS rates [including those of neutralizing antibodies, spike-specific immunoglobulin G (IgG), and RBD-specific IgG] in the elderly population after vaccination. Levels of all three of these antibody types, i.e., neutralizing, anti-S, and anti-RBD antibodies, increased significantly after vaccination, but there was no significant difference between them. Any number of vaccine doses was able to induce the production of antibodies in elderly people; however, the more frequent the inoculation doses, the higher the antibody titer levels were. There was not much difference in antibody titer levels between one or two doses, but the antibody titer levels increased significantly after three doses. With an increase in number of inoculation doses, the immune effect also increased correspondingly (65). Booster doses helped to increase the antibody titers and keep them stable. The standard fortification agent induces production of more antibody titers compared to the half-dose fortification agent and is more resistant to the delta variant than to the omicron variant (63).

Most of the approved COVID-19 vaccines, including the mRNA, recombinant adenovirus vector, inactivated coronavirus, and subunit vaccines, have been designed to elicit humoral and T-cell-mediated immune responses (70). In particular, COVID-19 vaccines can induce the production of anti-S, anti-RBD, and neutralizing antibodies against the spike protein of SARS-CoV-2 (71, 72), all of which bind to this spike protein and hinder its interaction with the angiotensin-converting enzyme 2 (ACE2) receptor (73). The viral entry of SARS-CoV-2 is facilitated by the interaction between its spike protein and the ACE2 receptor of the human host (74). Host protective antibodies induced by the COVID-19 vaccines hamper viral entry, the viral life cycle, and the pathogenicity of SARS-CoV-2. Although these antibody responses can be substantially boosted by two or three doses of the vaccines, the particular span of the duration of antibody responses remains unknown (75). The relevant findings of this meta-analysis once more suggests, in accordance with other reports on RCTs, that a second or booster dose of a vaccine triggers a considerable elevation in B-cell immune responses (57, 76). For this reason, the WHO recommends booster doses for priority vaccination groups, including elderly people, health workers, and other special groups (77, 78).

In this meta-analysis, it was found that, compared to the other types of vaccines, the nucleic acid vaccine produced the highest antibody titers in elderly people. This vaccine type induced the highest titer levels of spike-specific IgG, while the inactivated vaccine induced the lowest titer levels. Although the antibody titer levels of the elderly population after inoculation were lower than those of the younger population, the geometric mean ratios for antibodies were higher among the elderly population than among young people, which may have been a result of an insufficient number of studies involving the elderly population (64). In accordance with other reports, administration of the mRNA vaccine was associated with a significant increase in titer levels of neutralizing antibodies and in the antigen-specific production of IFN-γ, CD4^+^, and CD8^+^ T cells after the second vaccine dose (71, 72, 79). However, since only three articles involving this form of analysis were included, this conclusion may not be generalizable and may be limited to the vaccines included in the study. The analysis conducted in this study undoubtedly confirmed that the nucleic acid vaccine produced the best immune effect in the elderly population.

In the qualitative analysis, number of IFN-γ spot-forming cells (SFCs) and percentage of T-cell subsets were found to increase along with number of COVID-19 vaccine doses and time since inoculation among vaccinated elderly people (38, 44). The number of IFN-γ SFCs was slightly elevated on day 28 after the first dose of the viral vector vaccine compared to non-vaccination, but increased significantly after the second dose (44). After vaccination, the number of Th1 cells in the elderly population increased exponentially, while the number of Th2 cells fluctuated slightly with the increase in days since inoculation and number of doses (38, 66). Generally, the IFN response produced by alveolar macrophages, dendritic cells, natural killer cells, and inflammatory monocyte/macrophages is the primary antiviral innate immune signaling pathway (80). However, the number of articles reporting on RCTs examining T-cell responses and cytokine production after COVID-19 vaccination was insufficient, and these measures were not included in the analysis of protective immunity in the current study. Additionally, a number of older adults with diabetes or high blood pressure showed little difference in titer levels of neutralizing antibodies after vaccination when compared with healthy adults (69). Overall, the outcomes analysis of the retrospective studies showed that vaccination was effective in providing protection from SARS-CoV-2 infection, as well as in increasing the antibody positive conversion rate after vaccination.

In terms of the VS analysis, we found that vaccination will, to some extent, cause certain adverse reactions. The incidence of lAEs was higher than that of sAEs, and vaccination was not statistically associated with complications of geriatric diseases. Pain and fever were the most common lAEs and sAEs. Comparison of the pain and fever responses showed that pain was more prevalent than fever in the inoculated population. The frequency of local and systemic adverse reactions was higher after the first dose than after the second dose. In the meta-analysis, local adverse reactions after vaccination were more prevalent than systemic adverse reactions. The main reason for this may be that different elderly individuals perceive adverse reactions differently, and some elderly people are more sensitive to perceptions of physical injury (63, 64). Therefore, the previous assumption that inoculated groups are more sensitive to perceptions of physical injury is clearer.

In addition, we also found that the incidence of serious adverse reactions was very low. Some of the studies also reported on cardiovascular and cerebrovascular diseases arising in elderly people after vaccination, including vascular embolism, arrhythmias, and nervous system bleeding (81). Although severe adverse events have been reported at a rate of around five cases per one million in all those administered vaccine doses, this is extremely rare and is a very low rate (81). The data in the articles that covered such adverse reactions showed that the nucleic acid vaccine, compared with viral vector vaccination, is less likely to trigger cardiovascular and cerebrovascular diseases (68). Such reports of adverse events due to administration of COVID-19 vaccines have created vaccine hesitancy among elderly populations in many parts of the world. Millions of doses of the COVID-19 vaccines have already been administered around the world, and the safety of the vaccines has been frequently stressed by the many health authorities monitoring VS (82). It needs to be made abundantly clear to the elderly that the advantages of vaccination, which is the best method of controlling COVID-19 by preventing severe illness and related deaths, far outweigh any potential risks.

The present meta-analysis and systematic review have several limitations. First, this research was limited to studies published in Chinese and English, and there were some shortcomings in the inclusion of research published in other languages. Second, due to insufficient data, we were not able to conduct a subgroup analysis for comorbidities in the elderly population, such as diabetes, hypertension, and cancer. Third, in the study of outcome indicators, due to a lack of or inadequacy of relevant data, some studies were not comprehensive. For example, the analysis of vaccine effectiveness was not comprehensive enough due to insufficient data on elderly people after vaccination. Fourth, there was a large degree of heterogeneity among the included studies regarding VE, GMT, and VS. The results of the subgroup analyses should be interpreted with caution due to the diversity of influencing factors. Finally, there were not enough data to analyze long-term adverse effects after vaccination, and only short-term adverse effects, including slAEs and ssAEs, were analyzed in the current study. Moreover, in cases where the author could not be contacted to obtain detailed data, we used image extraction methods for data presented in images. Although there was no qualitative impact on the outcome indices, there were still some limitations in terms of the fine-grained data. In addition, we did not find enough data for a subgroup analysis of all types of vaccines for the elderly population. Nevertheless, this research provides some degree of insight into the effectiveness and safety of vaccination in the elderly population.

## Conclusions

This systematic review and meta-analysis have comprehensively synthesized the latest data on vaccine effectiveness, immunogenicity, and safety in older adults based on 22 RCTs. In the meta-analysis, we found that vaccination is more effective in preventing SARS-CoV-2 infection and in reducing the number of COVID-19-related deaths in elderly people. The effect of two doses s stronger than that of one dose. After vaccination, high AS rates are observed in the elderly population. With an increase in the number of inoculation doses received, antibody titer levels also increased among the older population, with the highest antibody titer levels in elderly people being induced by the nucleic acid vaccinein. Vaccination can produce certain adverse reactions in the elderly population, but their incidence is quite low. It needs to be made abundantly clear to elderly people that the advantages of vaccination, which is the best way to control COVID-19 by preventing severe illness and reducing related deaths, far outweigh any potential risks. However, more randomized clinical trials are needed to increase the certainty of the evidence and to draw more reliable conclusions.

## Data availability statement

The datasets presented in this study can be found in online repositories. The names of the repository/repositories and accession number(s) can be found in the article/**Supplementary Material**.

## Author contributions

KX and ZW performed the literature search and data extraction, and drafted the manuscript. MQ and YG were responsible for the quality assessment. NL, WX, and YZ conducted the statistical analysis. JW performed the literature search and data extraction. XM was responsible for the design and conceived the original idea. All authors contributed to the article and approved the submitted version.
