# Supplementary material for: A systematic review and meta-analysis of the effectiveness and safety of COVID-19 vaccination in older adults
Source: Front Immunol. 2023 Mar 3;14:1113156. doi: 10.3389/fimmu.2023.1113156 (PMC10020204; doi:10.3389/fimmu.2023.1113156)
Supplement: Supplementary file 1 [file DataSheet_1.pdf]

Supplementary material

**Appendix 1**	WHO approved 11 COVID-19 vaccines for the emergency use listing (EUL)	Page 1
**Appendix 2**	Status of COVID-19 Vaccines within WHO EUL/PQ evaluation process	Page 3
**Appendix 3**	Literature search strategy (PubMed, Web of science, Embase, Cochrane Library, Clinical trial.gov, Research Square, Opengray and Gray literature)	Page 5
**Appendix 4**	PRISMA checklist	Page 9
**Appendix 5**	Basic features of the retrospective literatures and outcomes of retrospective studies (including the quality assessment of evidence of RSs with GRADE system, the shape of funnel and forest plot of vaccine effectiveness, vaccine effectiveness (number of doses), vaccine effectiveness (vaccine type) and antibody seroconversion).	Page 11
**Appendix 6**	Basic features of the literatures included in the qualitative analysis.	Page 19
**Appendix 7**	The quality assessment of each RCT with RoB2 tool	Page 20
**Appendix 8**	The quality assessment of evidence of RCR literatures with GRADE system	Page 21
**Appendix 9**	The shape of the funnel plot of vaccine effectiveness (including number of doses, vaccine type), the shape of the funnel plot of GMT (including antibody type, vaccine type), and the shape of the funnel plot of solicited local adverse event and solicited systemic adverse event	Page 27
**Appendix 10**	Egger test	Page 30
**Appendix 11**	The shape of the forest plot of vaccine effectiveness (including number of doses, vaccine type), the shape of the forest plot of GMT (including antibody type, vaccine type), the shape of the forest plot of solicited local adverse event and solicited systemic adverse event	Page 31

Appendix 1. WHO approved 11 COVID-19 vaccines for the emergency use listing (EUL)

Vaccine	WHO EUL Holder	Country	First recommendation issued
**messenger-RNA (mRNA) vaccine**<br>COMIRNATY®<br>COVD-19 mRNA Vaccine (nucleoside modified)	BioNTech Manufacturing GmbH	Germany	31 Dec 2020
**Recombinant adenovirus vector vaccine**<br>COVID-19 Vaccine (ChAdOx1-S [recombinant])	AstraZeneca AB / SK Bioscience Co. Ltd<br>AstraZeneca AB	Sweden	15 Feb 2021
**Recombinant adenovirus vector vaccine**<br>**COVISHIELD™**<br>COVID-19 Vaccine (ChAdOx1-S [recombinant])	Serum Institute of India Pvt. Ltd	India	15 Feb 2021
**Recombinant adenovirus vector vaccine**<br>COVID-19 Vaccine (Ad26.COV2-S [recombinant])	Janssen–Cilag International NV	Belgium	12 Mar 2021
**messenger-RNA (mRNA) vaccine**<br>**SPIKEVAX**<br>COVID-19 mRNA Vaccine (nucleoside modified)	Moderna Biotech<br>ModernaTX, Inc	Spain	30 Apr 2021
**Inactivated vaccine**<br>Inactivated COVID-19 Vaccine	Beijing Institute of Biological Products Co.,	China	07 May 2021

(Vero Cell)	Ltd		
**Inactivated vaccine**<br>**CoronaVac**<br>COVID-19 Vaccine (Vero Cell), Inactivated	Sinovac Life Sciences Co., Ltd	China	01 Jun 2021
**Inactivated vaccine**<br>**COVAXIN®**<br>COVID-19 vaccine (Whole Virion Inactivated Corona Virus vaccine)	Bharat Biotech International Ltd	India	03 Nov 2021
**Recombinant S proteins subunit vaccine**<br>**COVOVAX™**<br>COVID-19 vaccine (SARS-CoV-2 rS Protein Nanoparticle [Recombinant])	Serum Institute of India Pvt. Ltd	India	17 Dec 2021
**Recombinant S proteins subunit vaccine**<br>**NUVAXOVID™**<br>COVID-19 vaccine (SARS-CoV-2 rS [Recombinant, adjuvanted])	Novavax CZ a.s.	Czech Republic	20 Dec 2021
**Recombinant adenovirus vector vaccine**<br>CONVIDECIA<br>COVID-19 Vaccine (Ad5-nCoV-S [Recombinant])	CanSino Biologics Inc.	China	19 May 2022

Appendix 2. Status of COVID-19 Vaccines within WHO EUL/PQ evaluation process

Status of COVID-19 Vaccines within WHO EUL/PQ evaluation process

	Manufacturer / WHO EUL holder	Name of Vaccine	NRA of Record	Platform	EOI accepted	Pre-submission meeting held	Dossier accepted for review*	Status of assessment**	Decision date***
1.	Pfizer BioNTech<br>BioNTech Manufacturing GmbH	BNT162b2/COMIRNATY Tozinameran (INN)	EMA	Nucleoside modified mRNA	✓	✓	✓	Finalized:<br><br>Additional sites:<br>– Baxter Oncology GmbH Germany (DP)<br>Novartis Switzerland<br>– Mibe (Dermapharm) Germany (DP)<br>– Delpharm, Saint-Remy FRANCE (DP)<br>– Sanofi-Aventis Deutschland GmbH Germany (DP)<br>– Siegfried Hameln GmbH, Germany (DP)<br>– Patheon Italia S.p.A, Italy (DP)<br>Catalent Agnani<br>Exela Pharma Sciences, LLC, NC<br>Sanofi-Aventis Deutschland GmbH (DP)<br><br>Diluent suppliers:<br>– Pfizer Perth, Australia<br>– Fresenius Kabi, USA<br>– Pfizer Manufacturing Belgium<br>Kwang Myung Pharm Co., Ltd.<br><br>Shelf life extension: 15 months at -70 to -90°C (PBS/Sucrose)<br>Shelf life extension: 12 months at -70 to -90°C PBS/Tris<br>Booster dose approved for adults 18 years of age and older<br>Age extension to adolescents 12-15<br>Age extension to children 5 – 11 years of age	31/12/2020<br><br>30/06/2021<br>08/07/2021<br>16/07/2021<br>17/09/2021<br>18/062021<br>11/11/2021<br>07/12/2021<br>21/01/2022<br>16/03/2022<br>12/09/2022<br><br>20/09/2021<br>20/09/2021<br>30/11/2021<br>14/01/2022<br><br>29/08/2022<br>18/05/2022<br>17/12/2021<br>08/09/2021<br>12/02/2022
			USFDA				✓	Finalized<br>Pharmacia & Upjohn, Kalamazoo (DP)<br>– PGS McPherson (DP)<br>– Exelead, Inc. Indianapolis USA	16/07/2021<br>16/07/2021<br>16/07/2021<br>30/09/2021
2.	AstraZeneca Oxford<br>AstraZeneca, AB	AZD1222 Vaxzevria	EMA	Recombinant ChAdOx1 adenoviral vector encoding the Spike protein antigen of the SARS-CoV-2.	✓	✓	✓	Core data finalized<br><br>Booster dose approved for adults 18 years of age and older	16 April 2021<br><br>19 July 2022
							✓	Finalized:<br>Additional sites:<br>– SK-Catalent<br>– Wuxi (DS)<br>– Chemo Spain<br>– Amylin Ohio US (DP)<br>– WuXi Biologics, Germany (DP)	16/04/2021<br>30/04/2021<br>30/04/2021<br>04/06/2021<br>23/07/2021<br>08/03/2022
3.			MFDS KOREA	Recombinant ChAdOx1 adenoviral vector encoding the Spike protein antigen of the SARS-CoV-2.	✓	✓	✓	Finalized	15 Feb 2021
4.			Japan MHLW/PMDA	Recombinant ChAdOx1 adenoviral vector encoding the Spike protein antigen of the SARS-CoV-2.	✓	✓	✓	Finalized<br>Additional sites:<br>Nipro Pharma Corporation Ise, Japan	09 July 2021<br><br>11 October 2021
5.			Australia TGA	Recombinant ChAdOx1 adenoviral vector encoding the Spike protein antigen of the SARS-CoV-2.	✓	✓	✓	Finalized<br>Additional site:<br>Siam Bioscience Co., Ltd Thailand	09 July 2021<br><br>11 October 2021
6.			COFEPRIS (Mexico)<br>ANMAT (Argentina)	Recombinant ChAdOx1 adenoviral vector encoding the Spike protein antigen of the SARS-CoV-2.	✓	✓	✓	Finalized	23 December 2021
7.	SERUM INSTITUTE OF INDIA PVT. LTD. Cyrus Poonawalla Group<br>Serum Institute of India Pvt. Ltd	Covishield (ChAdOx1_nCoV-19)	DCGI	Recombinant ChAdOx1 adenoviral vector encoding the Spike protein antigen of the SARS-CoV-2.	✓	✓	✓	Finalized<br>DS and DP Manjari Bk Pune	15 Feb 2021<br>12 Nov 2021
8.	Janssen Infectious Diseases & Vaccines<br>Janssen–Cilag International NV	Ad26.COV2.S	EMA	Recombinant, replication-incompetent adenovirus type 26 (Ad26) vectored vaccine encoding the (SARS-CoV-2) Spike (S) protein	✓	✓	✓	Core data finalized (US +NL sites)<br>- Additional sites: Aspen RSA (DP)<br>- Catalent Agnani Italy (DP)<br>- Grand River Aseptic Manufacturing Inc., USA<br>- MSD (Merck(, West Point/PA, USA (DP)<br>- Sanofi Pasteur France (DP)<br>- Biological E Ltd India (DP)<br><br>Storage conditions extension at 2-8 °C from 4,5 months to 11 months within the 24 months of shelf-life at -25°C to -15°C<br><br>Booster dose approved for adults 18 years of age and older	12 March 2021<br><br>25 June 2021<br>02 July 2021<br>17 sept 2021<br><br>05 Nov 2021<br>27 Jan 2022<br>07 July 2022<br><br>16/03/2022<br><br>25/03/2022
			DCGI	Recombinant, replication-incompetent adenovirus type 26 (Ad26) vectored vaccine encoding the (SARS-CoV-2) Spike (S) protein	✓	✓	✓	Ongoing	To be confirmed
9.	moderna<br>Moderna Biotech	mRNA-1273	EMA	mRNA-based vaccine encapsulated in lipid nanoparticle (LNP)	✓	✓	✓	Finalized<br><br>Shelf life extension to 09 months -20±5°C	30 April 2021<br><br>14 Feb 2022
			USFDA	mRNA-based vaccine encapsulated in lipid nanoparticle (LNP)	✓	✓	✓	Finalized<br>- ModernaTx. Norwood (DS)<br>- Catalent Indiana, LLC (DP)<br>- Lonza Biologics, Inc. Portsmouth, USA (DS)<br>- Baxter, Bloomington, USA (DP)	06 August 2021
			MFDS	mRNA-based vaccine encapsulated in lipid nanoparticle (LNP)	✓	✓	✓	Finalized	23 December 2021
10.	Sinopharm / BIBP[1]<br>Beijing Institute of Biological Products Co., Ltd. (BIBP)	SARS-CoV-2 Vaccine (Vero Cell), Inactivated (InCoV)	NMPA	Inactivated, produced in Vero cells	✓	✓	✓	Finalized<br>2 and 5 dose presentation (new manufacturing site)	07 May 2021<br>28 December 2021
11.	sinovac<br>Sinovac Life Sciences Co., Ltd.<br>Sinovac Life Sciences Co., Ltd.	COVID-19 Vaccine (Vero Cell), Inactivated/ Coronavac™	NMPA	Inactivated, produced in Vero cells	✓	✓	✓	Finalized<br>2 dose presentation	01 June 2021<br>30 September 2021
12.	BHARAT BIOTECH<br>Bharat Biotech, India	SARS-CoV-2 Vaccine, Inactivated (Vero Cell)/ COVAXIN	DCGI	Whole-Virion Inactivated Vero Cell	✓	✓	✓	Finalized	03 November 2021<br>SUPPLY OF VACCINE SUSPENDED

	Manufacturer / WHO EUL holder	Name of Vaccine	NRA of Record	Platform	EOI accepted	Pre-submission meeting held	Dossier accepted for review*	Status of assessment**	Decision date***
13.	SERUM INSTITUTE OF INDIA PVT. LTD. Cyrus Poonawalla Group	NVX-CoV2373/Covovax	DCGI	Recombinant nanoparticle prefusion spike protein formulated with Matrix-M™ adjuvant	✓		Rolling data started 21 September 2021	Finalized	17 December 2021
14.	NOVAVAX	NVX-CoV2373/Nuvaxovid	EMA	Recombinant nanoparticle prefusion spike protein formulated with Matrix-M™ adjuvant	✓	✓	Rolling data started 19 August 2021	Finalized<br><br>Additional sites:<br>SK Bioscience Co., Ltd., (DS)	20 December 2021<br><br>1/09/2022
15.	康希诺生物 CanSinoBIO	Ad5-nCoV	NMPA	Recombinant Novel Coronavirus Vaccine (Adenovirus Type 5 Vector)	✓	✓	Rolling data started 09 August 2021	Finalized	19 May 2022
16.	RUSSIAN DIRECT INVESTMENT FUND	Sputnik V	Russian NRA	Human Adenovirus Vector-based Covid-19 vaccine	Additional information submitted	Several meetings have been and continue to be held.	"Rolling" submission incomplete.	Process restarted, awaiting completion of rolling submission and CAPAs to last inspection	Anticipated date will be set once all data is submitted and follow-up of inspection observations completed.
17.	SANOFI	CoV2 preS dTM-AS03 vaccine	EMA	Recombinant, adjuvanted	✓	✓	Rolling data started 30 July 2021	Ongoing	To be confirmed
18.	Clover Biopharmaceuticals	SCB-2019	NMPA	Novel recombinant SARS-CoV-2 Spike (S)-Trimer fusion protein	✓	✓	Rolling data started 20 September	Ongoing	To be confirmed
19.	Zhifei Longcom, China	Recombinant Novel Coronavirus Vaccine （CHO Cell)	NMPA	Recombinant protein subunit	✓	✓	Rolling data started 28 March 2022	Ongoing	To be confirmed
20.	Shifa Pharmed - Barkat	CovIran× vaccine	Iran Food Drug Administration (IFDA)	Inactivated, produced in Vero cells	✓	✓	Rolling data started 3 August 2022	Ongoing	To be confirmed
21.	CIGB	Abdala	CECMED	Protein subunit	✓	✓	Rolling data started 7 June 2022	Ongoing	To be confirmed
22.	SK Bioscience	Nuvaxovid prefilled syringe	MFDS (RoKorea)	Recombinant nanoparticle prefusion spike protein formulated with Matrix-M™ adjuvant	✓	✓	Rolling data pending	Ongoing	To be confirmed
23.	Biological E	Corbevax	DCGI India	RBD antigen of SARS CoV-2 (Covid-19)	✓	✓	Rolling data started 10th of June	Ongoing	To be confirmed
24.	SK Bioscience	GBP510	MFDS (RoKorea)	Recombinant protein subunit	✓	✓	Rolling data started 7 September 2022	Ongoing	To be confirmed
25.	WestVac Biopharma	Recombinant COVID-19 Vaccine	NMPA China	Recombinant SARS-CoV-2 S-RBD protein	EOI under review				
26.	Nanogen	Nanocovax	Drug Administration of Vietnam	Recombinant Spike protein	EOI under review				
27.	Cinnagen	SpikoGen	Iran Food Drug Administration (IFDA)	Recombinant Protein	EOI under review				
28.	R-PHARM	Vaccine R-COVI	Russian NRA	Recombinant ChAdOx1 adenoviral vector encoding the Spike protein antigen of the SARS-CoV-2.	EOI under review				
29.	Arcturus Therapeutics	ARCT-154	Drug Administration of Vietnam	RNA Vaccine	EOI under review				
30.	Bio-Manguinhos/Fiocruz	AZD1222	ANVISA	Recombinant ChAdOx1 adenoviral vector encoding the Spike protein antigen of the SARS-CoV-2.	EOI under review				
31.	Vaxxinity	UB-612	FDA	Protein-peptide vaccine	EOI under review				
32.	Sinocelltech, Ltd	SCTV01C	NMPA	Recombinant Protein	EOI received				
33.	Razi Vaccine & Serum Research Institute	Razi Cov Pars Vaccine	Iran Food Drug Administration (IFDA)	Recombinant Protein	EOI received				
34.	Valneva	VLA2001	EMA	Inactivated	EOI received				
35.	Medigen	MVC-COV1901	TGA	CHO cell derived spike protein (Subunit)	EOI received				
36.	HIPRA	BIMERVAX	EMA	Recombinant Protein	EOI received				
37.	Stelis Biopharma Limited	AKS-452 Vaccine (AmbiVax -CTM)	DCGI India	Protein subunit	EOI received				
38.	PT Biofarma	SARS CoV-2 RBD	Badan Pom Indonesia	Recombinant Protein Vaccine	EOI received				
39.	Medicago	COVIFENZ®	Health Canada	Plant-based virus-like particle [VLP], recombinant, adjuvanted	Application withdrawn by applicant				
40.	CUREVAC	Zorecimeran (INN) concentrate and solvent for dispersion for injection; Company code: CVnCoV/CV07050101	EMA	mRNA-based vaccine encapsulated in lipid nanoparticle (LNP)	✓	Application withdrawn by manufacturer			
41.	Sinopharm / WIBP[2]	Inactivated SARS-CoV-2 Vaccine (Vero Cell)	NMPA	Inactivated, produced in Vero cells	✓	✓	Rolling data started 23 July 2021	Dossier withdrawn on 7 September 2022	
42.	Vector State Research Centre of Viralogy and Biotechnology	EpiVacCorona	Russian NRA	Peptide antigen	Letter received not EOI. Reply sent on 15/01/2021				
43.	IMBCAMS, China	SARS-CoV-2 Vaccine, Inactivated (Vero Cell)	NMPA	Inactivated	Not accepted, still under initial development				
44.	BioCubaFarma - Cuba	Soberana 01, Soberana 02 Soberana Plus	CECMED	SARS-CoV-2 spike protein conjugated chemically to meningococcal B or tetanus toxoid or Aluminum	Awaiting information on strategy and timelines for submission.				

1. Beijing Institute of Biological Products Co-Ltd
2. Wuhan Institute of Biological Products Co Ltd

* Dossier Submission dates: more than one date is possible because of the rolling submission approach. Dossier is accepted after screening of received submission.
**Status of assessment: 1. Under screening; 2. Under assessment; 3. Waiting responses from the applicant. 4. Risk-benefit decision 5. Final decision made
*** Anticipated decision date: this is only an estimate because it depends on when all the data is submitted under rolling submission and when all the responses to the assessors' questions are submitted.

Please send any questions you may have to: WHOEUL@who.int

Appendix 3. literature search strategy

(PubMed, Web of science, Embase, Cochrane Library, Clinical trial.gov, Research Square, Open gray and Gray literature)

Pubmed search strategy (before 1 October 2022)

("older adults"[Title/Abstract] OR "old people"[Title/Abstract] OR "old population"[Title/Abstract] OR "the aged"[Title/Abstract] OR "elder people"[Title/Abstract] OR "the elderly"[Title/Abstract] OR "older patients"[Title/Abstract] OR "aging"[Title/Abstract] OR "gerontology"[Title/Abstract]) AND 2020/01/01:2022/10/01[Date - Publication] AND (("vaccines"[MeSH Terms] OR "Vaccine"[Title/Abstract] OR "vaccin*"[Title/Abstract] OR "vaccination"[Title/Abstract]) AND 2020/01/01:2022/10/01[Date - Publication]) AND (("coronavirus"[MeSH Terms] OR "coronavirus"[Title/Abstract] OR "COVID-19"[Title/Abstract] OR "SARS-CoV-2"[Title/Abstract] OR "Variant strain"[Title/Abstract] OR "Delta variant"[Title/Abstract] OR "B.1.617.2"[Title/Abstract] OR "Omicron variant"[Title/Abstract] OR "B.1.1.529"[Title/Abstract]) AND 2020/01/01:2022/10/01[Date - Publication]) AND (("randomized controlled trial"[Publication Type] OR "controlled clinical trial"[Publication Type] OR "randomized"[Title/Abstract] OR "randomly"[Title/Abstract] OR "trial"[Title/Abstract] OR "groups"[Title/Abstract]) AND 2020/01/01:2022/10/01[Date - Publication]) AND 2020/01/01:2022/10/01[Date - Publication]

Embase search strategy (before 1 October 2022)

No.	Query Results
#30. #10 AND #14 AND #24 AND #29	107
#29. #28 OR #25 OR #26 OR #27	484,156
#28. 'trial':ab,ti AND [01-01-2020]/sd NOT [02-10-2022]/sd	232,599
#27. 'randomlized':ab,ti AND [01-01-2020]/sd NOT [02-10-2022]/sd	0
#26. 'randomly':ab,ti AND [01-01-2020]/sd NOT [02-10-2022]/sd	98,333
#25. 'random*':ab,ti AND [01-01-2020]/sd NOT [02-10-2022]/sd	380,943
#24. #15 OR #16 OR #17 OR #18 OR #19 OR #20 OR #21 OR #22 OR #23	121,855

#23. 'gerontology':ab,ti AND [01-01-2020]/sd NOT [02-10-2022]/sd 693

#22. 'aging':ab,ti AND [01-01-2020]/sd NOT [02-10-2022]/sd 54,509

#21. 'older patients':ab,ti AND [01-01-2020]/sd NOT [02-10-2022]/sd 15,999

#20. 'the elderly':ab,ti AND [01-01-2020]/sd NOT [02-10-2022]/sd 23,792

#19. 'elder people':ab,ti AND [01-01-2020]/sd NOT [02-10-2022]/sd 98

#18. 'the aged':ab,ti AND [01-01-2020]/sd NOT [02-10-2022]/sd 2,172

#17. 'old population':ab,ti AND [01-01-2020]/sd NOT [02-10-2022]/sd 352

#16. 'old people':ab,ti AND [01-01-2020]/sd NOT [02-10-2022]/sd 585

#15. 'older adults':ab,ti AND [01-01-2020]/sd NOT [02-10-2022]/sd 40,608

#14. #11 OR #12 OR #13 105,879

#13. 'vaccination':ab,ti AND [01-01-2020]/sd NOT [02-10-2022]/sd 50,107

#12. 'vaccine':ab,ti AND [01-01-2020]/sd NOT [02-10-2022]/sd 63,477

#11. 'vaccine'/exp AND [01-01-2020]/sd NOT [02-10-2022]/sd 77,983

#10. #1 OR #2 OR #3 OR #4 OR #5 OR #6 OR #7 OR #8 OR #9 303,325

#9. 'b.1.1.529':ab,ti AND [01-01-2020]/sd NOT [02-10-2022]/sd 638

#8. 'omicron variant':ab,ti AND [01-01-2020]/sd NOT [02-10-2022]/sd 1,617

#7. 'b.1.617.2':ab,ti AND [01-01-2020]/sd NOT [02-10-2022]/sd 888

#6. 'delta variant':ab,ti AND [01-01-2020]/sd NOT [02-10-2022]/sd 1,631

#5. 'coronavirinae'/exp AND [01-01-2020]/sd NOT [02-10-2022]/sd 87,637

#4. 'sars-cov-2':ab,ti AND [01-01-2020]/sd NOT [02-10-2022]/sd 88,744

#3. 'covid-19':ab,ti AND [01-01-2020]/sd NOT [02-10-2022]/sd 259,120

#2. 'coronavirus':ab,ti AND [01-01-2020]/sd NOT [02-10-2022]/sd 91,087

#1. 'coronavirinae'/exp AND [01-01-2020]/sd NOT [02-10-2022]/sd 87,637

Cochrane Library search strategy (before 1 October 2022)

#1 MeSH descriptor: [Vaccines] explode all trees 14266

#2 (Vaccine):ti,ab,kw OR (vaccination):ti,ab,kw 28005

#3 (“the elderly”):ti,ab,kw OR (“aging”):ti,ab,kw OR (“gerontology”):ti,ab,kw 26200

#4 (“older adults”):ti,ab,kw OR (“old people”):ti,ab,kw OR (“old population”):ti,ab,kw OR (“the aged”):ti,ab,kw OR (“elder people”):ti,ab,kw 22386

#5 #1 OR #2 28655

#6 #3 OR #4 43214

#7 MeSH descriptor: [Coronavirus] explode all trees 1207

#8 (“coronavirus”):ti,ab,kw OR (“COVID-19”):ti,ab,kw OR (“SARS-CoV-2”):ti,ab,kw 14306

#9 (“Variant strain”):ti,ab,kw OR (“Delta variant”):ti,ab,kw OR (“B.1.617.2”):ti,ab,kw OR (“Omicron variant”):ti,ab,kw OR (“B.1.1.529”):ti,ab,kw 103

#10 #7 OR #8 14313

#11 #9 OR #10 14322

#12 #5 AND #11 1827

#13 #6 AND #12 77

Web of science search strategy (before 1 October 2022)

TS=(randomized controlled trial OR control* clinicalkey trial OR randomized OR randomly OR trial) AND

KP=(coronavirus OR COVID-19 OR SARS-CoV-2 OR Variant strain OR Delta variant OR B.1.617.2 OR Omicron variant OR B.1.1.529) AND

KP=(Vaccine* OR vaccination) AND

TS=(older adults OR old people OR old population OR the aged OR elder people OR the elderly OR aging OR gerontology OR older patients)

AND 2020-01-01---2022-10-01

AND English

Clinical trial.gov (before 1 October 2022)

Vaccines AND elderly | Studies With Results | COVID -19 | Older Adult | Phase 1, 2, 3, 4 | Results first posted from 01/01/2020 to 10/01/2022

Research Square (before 1 October 2022)

Search: vaccine AND elderly
Journals & Platforms: Research square
Publication Status: Posted
Article Type: Research article
COVID-19 Preprints Only

Posted after 2020/01/01
Posted before 2022/10/01

Open gray (before 1 October 2022)
(randomized controlled trial OR control* clinicalkey trial OR randomized OR randomly OR trial) AND(coronavirus OR COVID-19 OR SARS-CoV-2 OR Variant strain OR Delta variant OR B.1.617.2 OR Omicron variant OR B.1.1.529) AND(Vaccine* OR vaccination) AND(older adults OR old people OR old population OR the aged OR elder people OR the elderly OR aging OR gerontology OR older patients)

Gray literature (before 1 October 2022)
(randomized controlled trial OR control* clinicalkey trial OR randomized OR randomly OR trial) AND(coronavirus OR COVID-19 OR SARS-CoV-2 OR Variant strain OR Delta variant OR B.1.617.2 OR Omicron variant OR B.1.1.529) AND(Vaccine* OR vaccination) AND(older adults OR old people OR old population OR the aged OR elder people OR the elderly OR aging OR gerontology OR older patients)

Summary
PubMed =306; Embase =107; Cochrane Library =77; Web of science=100; Clinical trial.gov =13; Research Square=657; Open gray=0; Gray literature=0

Appendix 4. PRISMA checklist

Section/topic	Item No	Checklist item	Reported on page No
**Title**			
Title	1	Identify the report as a systematic review, meta-analysis, or both	1
**Abstract**			
Structured summary	2	Provide a structured summary including, as applicable, background, objectives, data sources, study eligibility criteria, participants, interventions, study appraisal and synthesis methods, results, limitations, conclusions and implications of key findings, systematic review registration number	2
**Introduction**			
Rationale	3	Describe the rationale for the review in the context of what is already known	Introduction
Objectives	4	Provide an explicit statement of questions being addressed with reference to participants, interventions, comparisons, outcomes, and study design (PICOS)	Introduction
**Methods**			
Protocol and registration	5	Indicate if a review protocol exists, if and where it can be accessed (such as web address), and, if available, provide registration information including registration number	No
Eligibility criteria	6	Specify study characteristics (such as PICOS, length of follow-up) and report characteristics (such as years considered, language, publication status) used as criteria for eligibility, giving rationale	Methods
Information sources	7	Describe all information sources (such as databases with dates of coverage, contact with study authors to identify additional studies) in the search and date last searched	Methods
Search	8	Present full electronic search strategy for at least one database, including any limits used, such that it could be repeated	Appendix 3
Study selection	9	State the process for selecting studies (that is, screening, eligibility, included in systematic review, and, if applicable, included in the meta-analysis)	Methods
Data collection process	10	Describe method of data extraction from reports (such as piloted forms, independently, in duplicate) and any processes for obtaining and confirming data from investigators	Methods
Data items	11	List and define all variables for which data were sought (such as PICOS, funding sources) and any assumptions and simplifications made	Methods
Risk of bias in individual studies	12	Describe methods used for assessing risk of bias of individual studies (including specification of whether this was done at the study or outcome level), and how this information is to be used in any data synthesis	Methods
Summary measures	13	State the principal summary measures (such as risk ratio, difference in means).	Methods
Synthesis of results	14	Describe the methods of handling data and combining results of studies, if done, including measures of consistency (such as I^2 statistic) for each meta-analysis	Methods
Risk of bias across studies	15	Specify any assessment of risk of bias that may affect the cumulative evidence (such as publication bias, selective reporting within studies)	Methods
Additional analyses	16	Describe methods of additional analyses (such as sensitivity or subgroup analyses, meta-regression), if done, indicating which were pre-specified	Methods
**Results**			
Study selection	17	Give numbers of studies screened, assessed for eligibility, and included in the review, with reasons for exclusions at each stage, ideally with a flow diagram	Figure 1
Study	18	For each study, present characteristics for which data were extracted (such as study size, PICOS, follow-up	Table 1

Section/topic	Item No	Checklist item	Reported on page No
characteristics		period) and provide the citations	
Risk of bias within studies	19	Present data on risk of bias of each study and, if available, any outcome-level assessment (see item 12).	Figure 2, Appendix 4
Results of individual studies	20	For all outcomes considered (benefits or harms), present for each study (a) simple summary data for each intervention group and (b) effect estimates and confidence intervals, ideally with a forest plot	Figure 4-7
Synthesis of results	21	Present results of each meta-analysis done, including confidence intervals and measures of consistency	Figures 4-7
Risk of bias across studies	22	Present results of any assessment of risk of bias across studies (see item 15)	Figure 3
Additional analysis	23	Give results of additional analyses, if done (such as sensitivity or subgroup analyses, meta-regression) (see item 16)	Table 2-3, Figures 8, Appendix 5-7
**Discussion**			
Summary of evidence	24	Summarise the main findings including the strength of evidence for each main outcome; consider their relevance to key groups (such as health care providers, users, and policy makers)	Discussion
Limitations	25	Discuss limitations at study and outcome level (such as risk of bias), and at review level (such as incomplete retrieval of identified research, reporting bias)	Discussion
Conclusions	26	Provide a general interpretation of the results in the context of other evidence, and implications for future research	Discussion
**Funding**			
Funding	27	Describe sources of funding for the systematic review and other support (such as supply of data) and role of funders for the systematic review	Page 26

Appendix 5. Basic features of the retrospective literatures and outcomes of retrospective study analysis (including the quality assessment of evidence of RSs with GRADE system, the shape of funnel and forest plot of vaccine effectiveness, vaccine effectiveness (number of doses), vaccine effectiveness (vaccine type) and antibody seroconversion).

Table 5-1. Basic features of the retrospective literatures

First author	Country	Study design	Vaccine name	Vaccine type	Number of doses	Research quantum (V/C)	Age	Gender (M/F)	NOS score
Bag Soytas R et al. (56)	Turkey	RS	CoronaVac, Pfizer-BioNTech	IV	Three doses	81	⩾60	48/33	5
San Román J et al. (57)	Spain	RS	BNT162b2	NAV	Two doses	1218	83.7(12.1)	351/867	4
Schultz BM et al. (58)	Chile	RS	CoronaVac	IV	Two doses	42	⩾60	NA	4
Arregocés-Castillo L et al. (59)	Colombia	RS	Ad26.COV2-S, BNT162b2, ChAdOx1 nCoV-19, CoronaVac	IV, VVV, NAV	Two doses	1414147/1414147	63-75	129252/1535764	3
Meyer M et al. (60)	France	RS	BNT162b2	NAV	Two doses	34/32	79-92	18/48	3
Haas EJ et al. (61)	Israel	RS	BNT162b2	NAV	Two doses	1015620/112345	65-85	NA	2
Nunes B et al. (62)	Portugal	RS	BNT162b2, mRNA-1273	NAV	Two doses	1187029/152280	65-110	588456/768167	3

RS, retrospective study; IV, Inactivated vaccine; VVV, Viral vector vaccines; NAV, Nucleic acid vaccine; V/C, vaccine/control or placebo control; M/F, male/female;

Table 5-2 The quality assessment of evidence of retrospective studies with GRADE system

Quality assessment							No of patients		Effect		Quality	Importance
No of studies	Design	Risk of bias	Inconsistency	Indirectness	Imprecision	Other considerations	Vaccine effectiveness	Control	Relative (95% CI)	Absolute		
**Vaccine effectiveness**												
15	observational studies	no serious risk of bias	no serious inconsistency	no serious indirectness	no serious imprecision	strong association	4311/9264883 (0%)	19709/3582266 (0.6%)	OR 0.05 (0.02 to 0.13)	5 fewer per 1000 (from 5 fewer to 5 fewer)	ÅÅÅO MODERATE	CRITICAL
								0.5%		5 fewer per 1000 (from 4 fewer to 5 fewer)		
**Vaccine effectiveness (Number of doses)**												
15	observational studies	no serious risk of bias	no serious inconsistency	no serious indirectness	no serious imprecision	strong association	4311/9264883 (0%)	19709/3582266 (0.6%)	OR 0.05 (0.02 to 0.13)	5 fewer per 1000 (from 5 fewer to 5 fewer)	ÅÅÅO MODERATE	IMPORTANT
								0.5%		5 fewer per 1000 (from 4 fewer to 5 fewer)		
**Vaccine effectiveness (Number of doses) - One dose**												
2	observational studies	no serious risk of bias	no serious inconsistency	no serious indirectness	no serious imprecision	none	36/129994 (0%)	417/129994 (0.3%)	OR 0.11 (0.03 to 0.44)	3 fewer per 1000 (from 2 fewer to 3 fewer)	ÅÅOO LOW	IMPORTANT

								0.3%		3 fewer per 1000 (from 2 fewer to 3 fewer)		
**Vaccine effectiveness (Number of doses) - Two dose**												
13	observational studies	no serious risk of bias	no serious inconsistency	no serious indirectness	no serious imprecision	none	4275/9134889 (0%)	19292/3452272 (0.6%)	OR 0.05 (0.02 to 0.13)	5 fewer per 1000 (from 5 fewer to 5 fewer)	ÅÅOO LOW	IMPORTANT
								0.5%		5 fewer per 1000 (from 4 fewer to 5 fewer)		
**Vaccine effectiveness (Vaccine type)**												
15	observational studies	no serious risk of bias	no serious inconsistency	no serious indirectness	no serious imprecision	strong association	4311/9264883 (0%)	19709/3582266 (0.6%)	OR 0.05 (0.02 to 0.13)	5 fewer per 1000 (from 5 fewer to 5 fewer)	ÅÅÅO MODERATE	IMPORTANT
								0.5%		5 fewer per 1000 (from 4 fewer to 5 fewer)		
**Vaccine effectiveness (Vaccine type) - Inactivated vaccine**												
2	observational studies	no serious risk of bias	no serious inconsistency	no serious indirectness	no serious imprecision	none	2127/1366568 (0.2%)	4389/1366568 (0.3%)	OR 0.55 (0.35 to 0.84)	1 fewer per 1000 (from 1 fewer to 2 fewer)	ÅÅOO LOW	IMPORTANT
								0.3%		1 fewer per 1000 (from 0 fewer to 2 fewer)		
**Vaccine effectiveness (Vaccine type) - Nucleic acid vaccine**												

9	observational studies	no serious risk of bias	no serious inconsistency	no serious indirectness	no serious imprecision	none	2102/7236861 (0%)	13196/1554244 (0.8%)	OR 0.03 (0.02 to 0.04)	8 fewer per 1000 (from 8 fewer to 8 fewer)	ÅÅOO LOW	IMPORTANT
								1%		10 fewer per 1000 (from 10 fewer to 10 fewer)		
**Vaccine effectiveness (Vaccine type) - Viral vector vaccine**												
4	observational studies	no serious risk of bias	no serious inconsistency	no serious indirectness	no serious imprecision	none	82/661454 (0%)	2124/661454 (0.3%)	OR 0.06 (0.02 to 0.17)	3 fewer per 1000 (from 3 fewer to 3 fewer)	ÅÅOO LOW	IMPORTANT
								0.3%		3 fewer per 1000 (from 2 fewer to 3 fewer)		

**Quality assessment**							**No of patients**		**Effect**		**Quality**	**Importance**
**No of studies**	**Design**	**Risk of bias**	**Inconsistency**	**Indirectness**	**Imprecision**	**Other considerations**	**GMT**	**Control**	**Relative (95% CI)**	**Absolute**		
**Seroconversion**												
3	observational studies	no serious risk of bias	no serious inconsistency	no serious indirectness	no serious imprecision	strong association	-	-	-	OR 29.44 (21.93 to 39.51)-	ÅÅÅO MODERATE	CRITICAL
								0%				

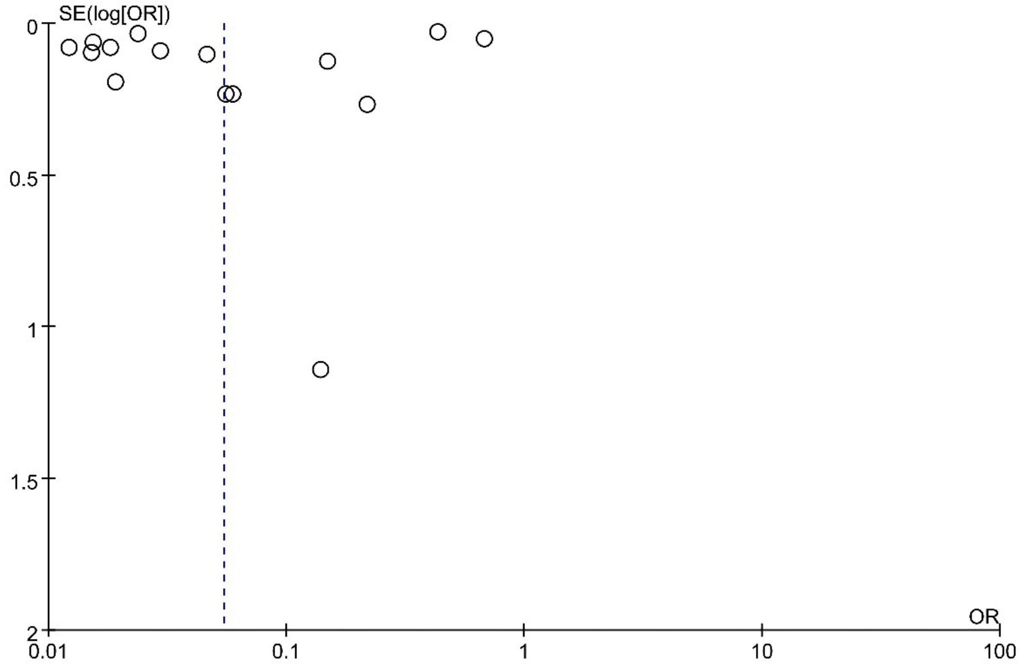

Figure 5-1 The shape of the funnel plot of vaccine effectiveness of retrospective studies

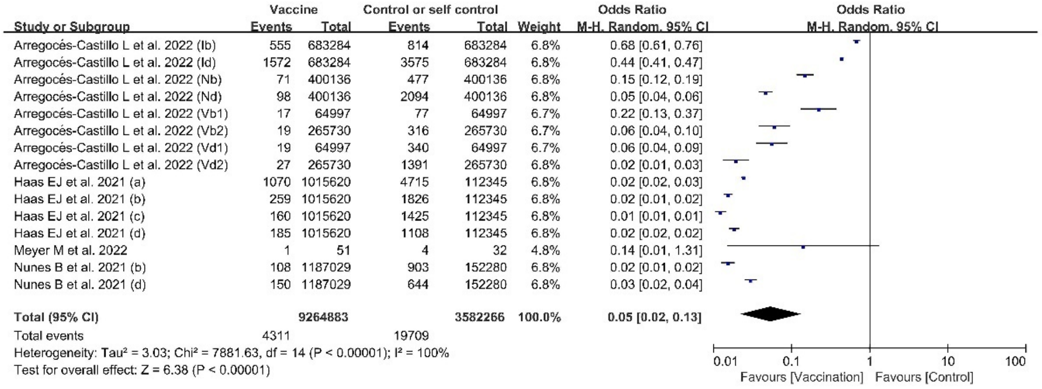

Figure 5-2 The shape of the forest plot of vaccine effectiveness of retrospective studies

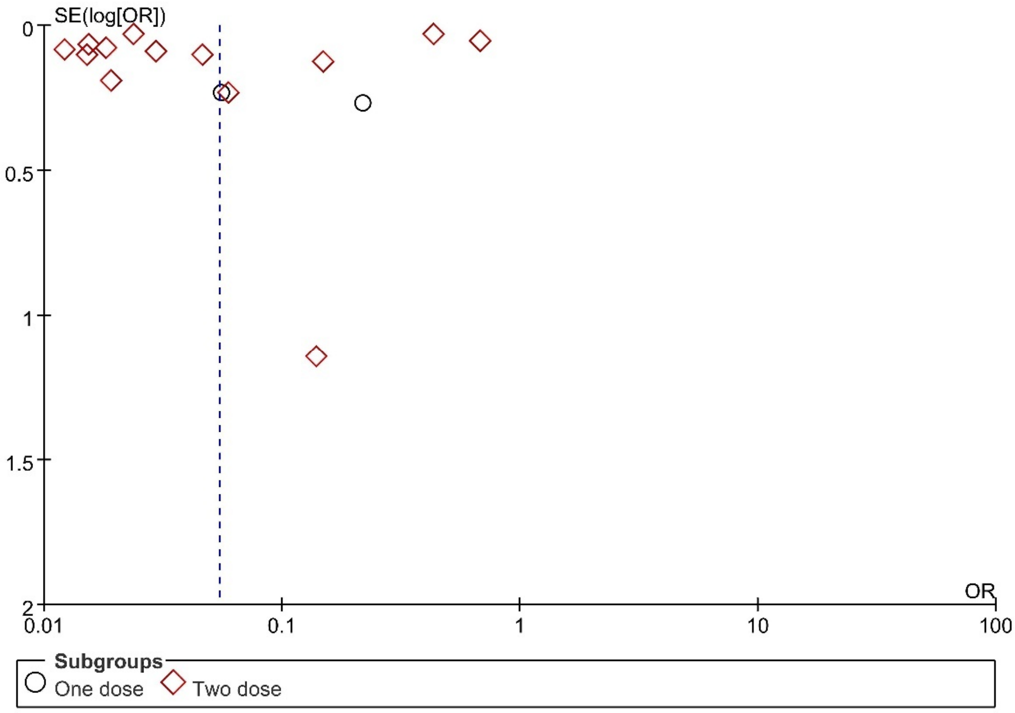

Figure 5-3 The shape of the funnel plot of vaccine effectiveness (number of doses) of retrospective studies

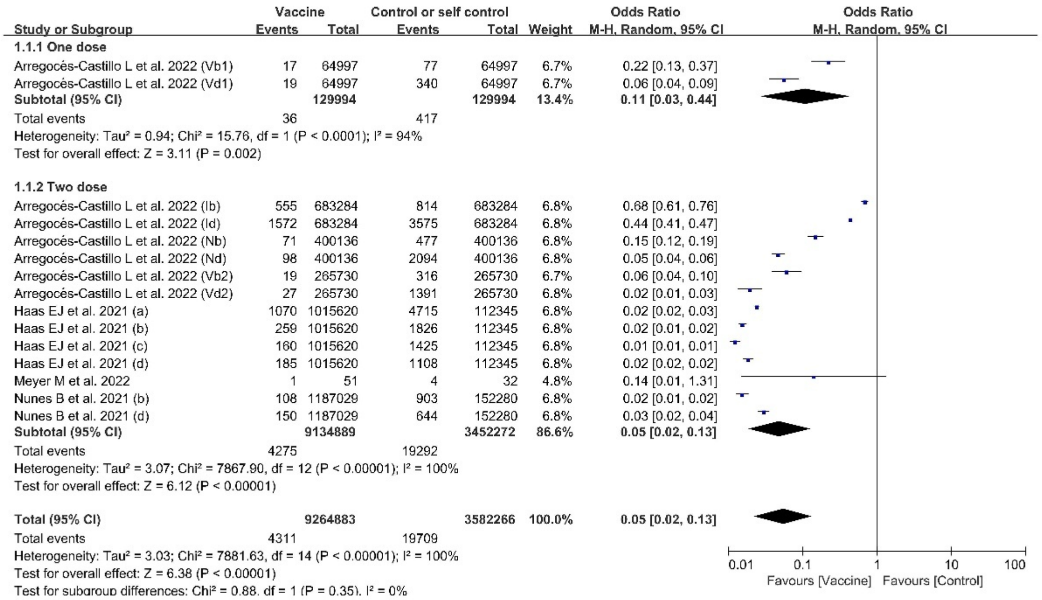

Study or Subgroup	Vaccine Events	Vaccine Total	Control or self control Events	Control or self control Total	Weight	Odds Ratio M-H, Random, 95% CI
**1.1.1 One dose**						
Arregocés-Castillo L et al. 2022 (Vb1)	17	64997	77	64997	6.7%	0.22 [0.13, 0.37]
Arregocés-Castillo L et al. 2022 (Vd1)	19	64997	340	64997	6.7%	0.06 [0.04, 0.09]
**Subtotal (95% CI)**		**129994**		**129994**	**13.4%**	**0.11 [0.03, 0.44]**
Total events	36		417			
Heterogeneity: Tau² = 0.94; Chi² = 15.76, df = 1 (P < 0.0001); I² = 94%						
Test for overall effect: Z = 3.11 (P = 0.002)						
**1.1.2 Two dose**						
Arregocés-Castillo L et al. 2022 (Ib)	555	683284	814	683284	6.8%	0.68 [0.61, 0.76]
Arregocés-Castillo L et al. 2022 (Id)	1572	683284	3575	683284	6.8%	0.44 [0.41, 0.47]
Arregocés-Castillo L et al. 2022 (Nb)	71	400136	477	400136	6.8%	0.15 [0.12, 0.19]
Arregocés-Castillo L et al. 2022 (Nd)	98	400136	2094	400136	6.8%	0.05 [0.04, 0.06]
Arregocés-Castillo L et al. 2022 (Vb2)	19	265730	316	265730	6.7%	0.06 [0.04, 0.10]
Arregocés-Castillo L et al. 2022 (Vd2)	27	265730	1391	265730	6.8%	0.02 [0.01, 0.03]
Haas EJ et al. 2021 (a)	1070	1015620	4715	112345	6.8%	0.02 [0.02, 0.03]
Haas EJ et al. 2021 (b)	259	1015620	1826	112345	6.8%	0.02 [0.01, 0.02]
Haas EJ et al. 2021 (c)	160	1015620	1425	112345	6.8%	0.01 [0.01, 0.01]
Haas EJ et al. 2021 (d)	185	1015620	1108	112345	6.8%	0.02 [0.02, 0.02]
Meyer M et al. 2022	1	51	4	32	4.8%	0.14 [0.01, 1.31]
Nunes B et al. 2021 (b)	108	1187029	903	152280	6.8%	0.02 [0.01, 0.02]
Nunes B et al. 2021 (d)	150	1187029	644	152280	6.8%	0.03 [0.02, 0.04]
**Subtotal (95% CI)**		**9134889**		**3452272**	**86.6%**	**0.05 [0.02, 0.13]**
Total events	4275		19292			
Heterogeneity: Tau² = 3.07; Chi² = 7867.90, df = 12 (P < 0.00001); I² = 100%						
Test for overall effect: Z = 6.12 (P < 0.00001)						
**Total (95% CI)**		**9264883**		**3582266**	**100.0%**	**0.05 [0.02, 0.13]**
Total events	4311		19709			
Heterogeneity: Tau² = 3.03; Chi² = 7881.63, df = 14 (P < 0.00001); I² = 100%						
Test for overall effect: Z = 6.38 (P < 0.00001)						
Test for subgroup differences: Chi² = 0.88, df = 1 (P = 0.35), I² = 0%						

Figure 5-4 The shape of the forest plot of vaccine effectiveness (number of doses) of retrospective studies

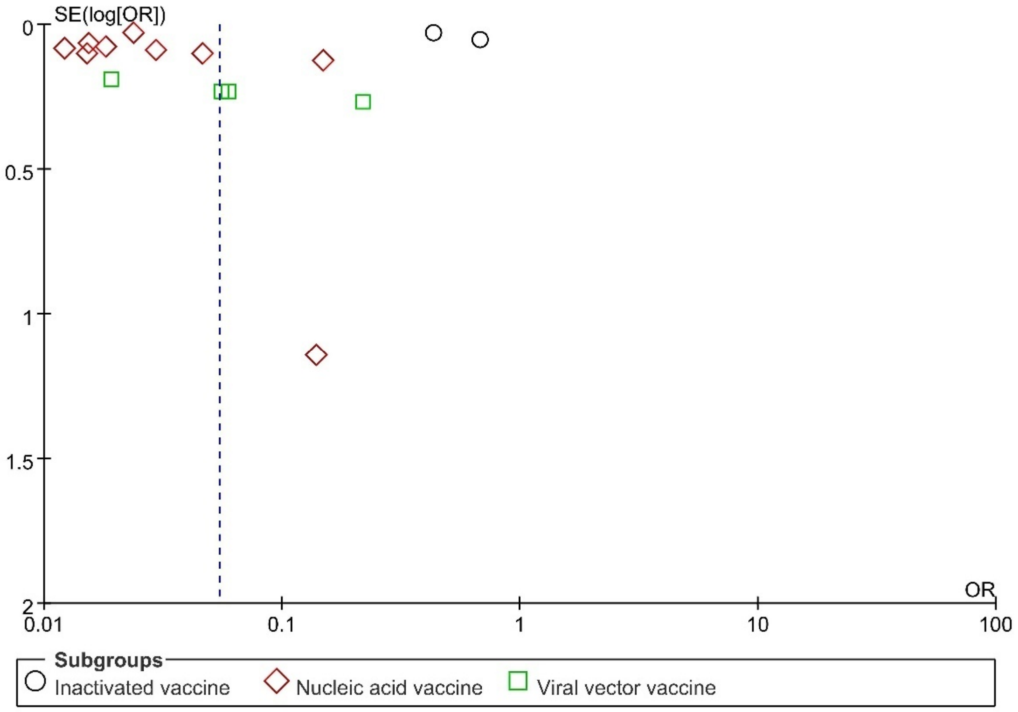

Figure 5-5 The shape of the funnel plot of vaccine effectiveness (vaccine type) of retrospective studies

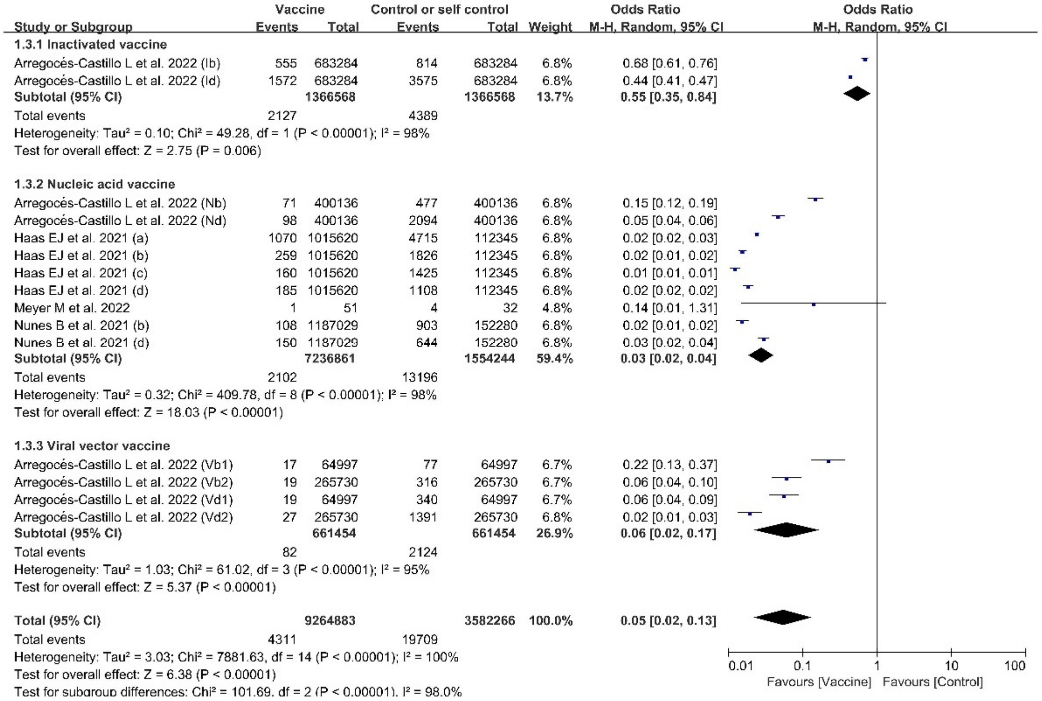

Figure 5-6 The shape of the forest plot of vaccine effectiveness (vaccine type) of retrospective studies

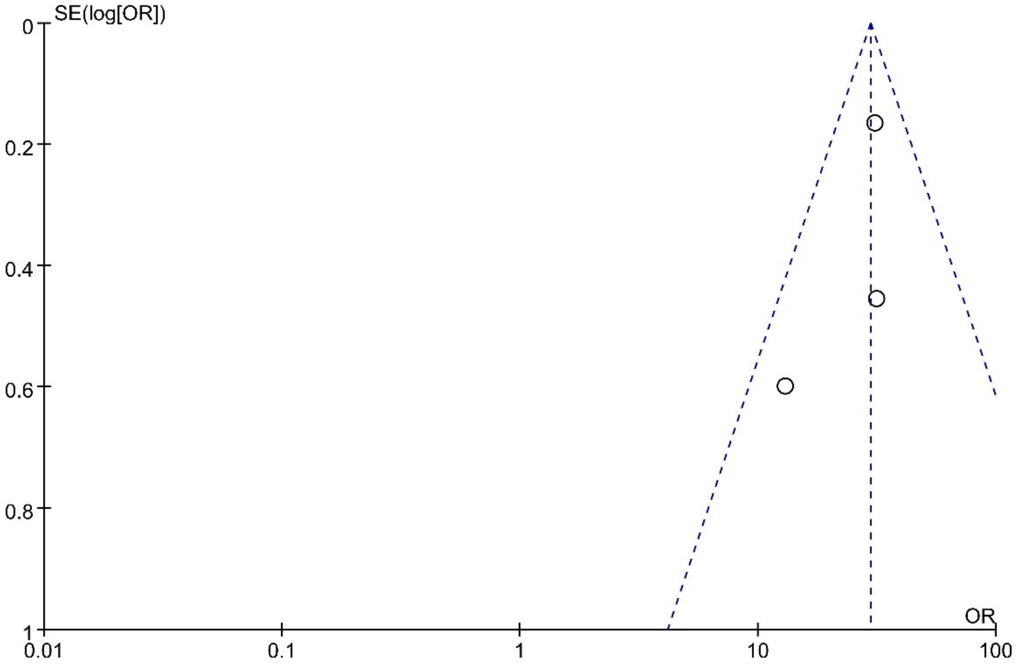

Figure 5-7 The shape of the funnel plot of antibody seroconversion rate of retrospective studies

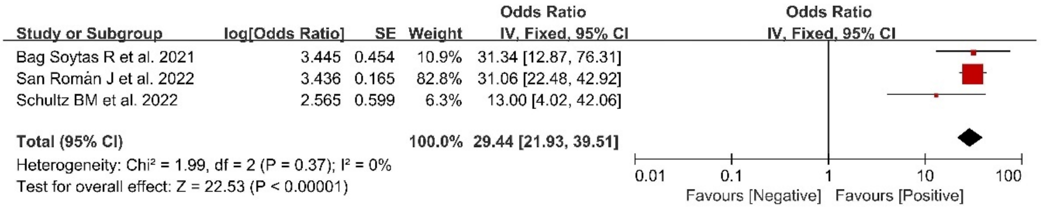

Figure 5-8 The shape of the forest plot of antibody seroconversion rate of retrospective studies

Appendix 6. Basic features of the literatures included in the qualitative analysis.

First author	Country	Vaccine name	Vaccine type	Number of doses	Research quantum (V/C)	Age	Gender (M/F)	RoB2	NOS score
Jose M et al.(63)	USA	ChAdOx1	VVV	Two doses	5/12	> 60	4/13	L	3
Nantanee R et al.(64)	Thailand	BNT162b2	NAV	Three doses	50/50	⩾ 60	39/61	S	4
Costa Clemens SA et al.(65)	Brazil	Ad26.COV2-S, BNT162b2, ChAdOx1 nCoV-19, CoronaVac	VVV, NAV, IV	One dose	1205	> 61	476/729	L	2
Sridhar S et al.(66)	USA	CoV2 preS dTM	SV	Two doses	721	≥60	362/359	L	3
Anderson EJ et al.(67)	USA	mRNA-1273	NAV	Two doses	40	> 56	19/21	S	2
Choi YY et al.(68)	Korea	BNT162b2	NAV	Two doses	5446	≥75	2418/3038	L	3
Cari L et al.(69)	Italy	BNT162b2, ChAdOx1 nCoV-19, Ad26.COV2.S	NAV, VVV	NA	NA	> 64	NA	S	1
Zhang Y rt al.(70)	China	BBIBP-CorV	IV	Two doses	327/329/296/480	⩾60	661/752	L	3

IV, Inactivated vaccine; SV, Subunit vaccine; VVV, Viral vector vaccines; NAV, Nucleic acid vaccine; V/C, vaccine/control or placebo control; M/F, male/female;

Appendix 7. The quality assessment of each study (n=22) with RoB2 tool

	Randomization process	Deviations from intended interventions	Mising outcome data	Measurement of the outcome	Selection of the reported result	Overall Bias
Low risk	20	22	22	22	21	19
Some concerns	2	0	0	0	1	3
High risk	0	0	0	0	0	0

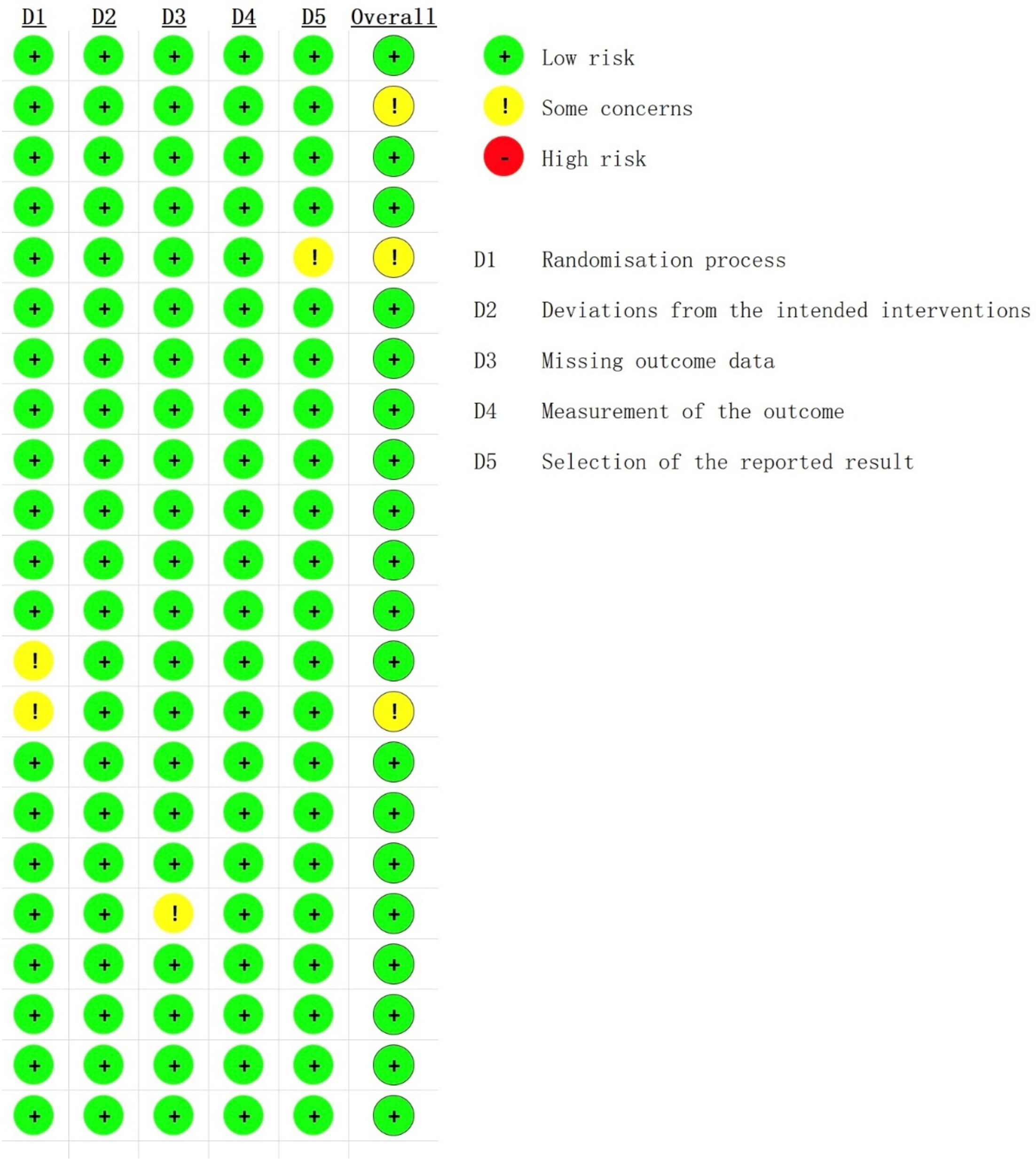

Appendix 8. The quality assessment of evidence of RCT literatures with GRADE system

Quality assessment							No of patients		Effect		Quality	Importance
**No of studies**	**Design**	**Risk of bias**	**Inconsistency**	**Indirectness**	**Imprecision**	**Other considerations**	**Vaccine effectiveness**	**Control**	**Relative (95% CI)**	**Absolute**		
**Vaccine effectiveness (Infection after vaccination)**												
3	randomised trials	no serious risk of bias	no serious inconsistency	no serious indirectness	no serious imprecision	strong association	2640/851047 (0.3%)	7121/849100 (0.8%)	OR 0.38 (0.23 to 0.65)	5 fewer per 1000 (from 3 fewer to 6 fewer)	ÅÅÅÅ HIGH	CRITICAL
								0.8%		5 fewer per 1000 (from 3 fewer to 6 fewer)		
**Vaccine effectiveness (Hospitalized or admitted to ICU after vaccination)**												
2	randomised trials	no serious risk of bias	no serious inconsistency	no serious indirectness	no serious imprecision	strong association[1]	162/12806 (1.3%)	168/12680 (1.3%)	OR 0.95 (0.77 to 1.19)	1 fewer per 1000 (from 3 fewer to 2 more)	ÅÅÅÅ HIGH	CRITICAL
								1.3%		1 fewer per 1000 (from 3 fewer to 2 more)		
**Vaccine effectiveness (Died after vaccination)**												
3	randomised trials	no serious risk of bias	no serious inconsistency	no serious indirectness	no serious imprecision	strong association[1]	77/847738 (0%)	534/847896 (0.1%)	OR 0.15 (0.12 to 0.19)	1 fewer per 1000 (from 1 fewer to 1 fewer)	ÅÅÅÅ HIGH	CRITICAL
								1.8%		15 fewer per 1000 (from 15 fewer to 16 fewer)		
**Vaccine effectiveness (Number of doses)**												
8	randomised trials	no serious risk of bias	no serious inconsistency	no serious indirectness	no serious imprecision	none	2879/1711591 (0.2%)	7823/1709676 (0.5%)	OR 0.45 (0.28 to 0.7)	3 fewer per 1000 (from 1 fewer to 3 fewer)	ÅÅÅÅ HIGH	IMPORTANT
								1.3%		7 fewer per 1000 (from 4 fewer to 9 fewer)		
**Vaccine effectiveness (Number of doses) - One dose**												
4	randomised trials	no serious risk of bias	no serious inconsistency	no serious indirectness	no serious imprecision	none	677/19255 (3.5%)	973/19133 (5.1%)	OR 0.81 (0.56 to 1.17)	9 fewer per 1000 (from 22 fewer to 8 more)	ÅÅÅÅ HIGH	IMPORTANT

								1.7%		3 fewer per 1000 (from 7 fewer to 3 more)		
**Vaccine effectiveness (Number of doses) - Two dose**												
4	randomised trials	no serious risk of bias	no serious inconsistency	no serious indirectness	no serious imprecision	none	2202/1692336 (0.1%)	6850/1690543 (0.4%)	OR 0.23 (0.11 to 0.45)	3 fewer per 1000 (from 2 fewer to 4 fewer)	ÅÅÅÅ HIGH	IMPORTANT
								0.8%		6 fewer per 1000 (from 4 fewer to 7 fewer)		
**Vaccine effectiveness (Vaccine type)**												
8	randomised trials	no serious risk of bias	no serious inconsistency	no serious indirectness	no serious imprecision	none	2879/1711591 (0.2%)	7823/1709676 (0.5%)	OR 0.45 (0.28 to 0.7)	3 fewer per 1000 (from 1 fewer to 3 fewer)	ÅÅÅÅ HIGH	IMPORTANT
								1.3%		7 fewer per 1000 (from 4 fewer to 9 fewer)		
**Vaccine effectiveness (Vaccine type) - Inactivated vaccine**												
2	randomised trials	no serious risk of bias	no serious inconsistency	no serious indirectness	no serious imprecision	none	0/68 (0%)	4/226 (1.8%)	OR 0.69 (0.08 to 6)	5 fewer per 1000 (from 16 fewer to 80 more)	ÅÅÅÅ HIGH	IMPORTANT
								1.8%		6 fewer per 1000 (from 17 fewer to 81 more)		
**Vaccine effectiveness (Vaccine type) - Nucleic acid vaccine**												
2	randomised trials	no serious risk of bias	no serious inconsistency	no serious indirectness	no serious imprecision	none	2197/1688618 (0.1%)	6834/1688618 (0.4%)	OR 0.22 (0.1 to 0.5)	3 fewer per 1000 (from 2 fewer to 4 fewer)	ÅÅÅÅ HIGH	IMPORTANT
								0.4%		3 fewer per 1000 (from 2 fewer to 4 fewer)		
**Vaccine effectiveness (Vaccine type) - Viral vector vaccine**												
4	randomised trials	no serious risk of bias	no serious inconsistency	no serious indirectness	no serious imprecision	none	682/22905 (3%)	985/20832 (4.7%)	OR 0.69 (0.46 to 1.05)	14 fewer per 1000 (from 25 fewer to 2 more)	ÅÅÅÅ HIGH	IMPORTANT
								1.3%		4 fewer per 1000 (from 7 fewer to 1 more)		

Quality assessment							No of patients		Effect		Quality	Importance
No of studies	Design	Risk of bias	Inconsistency	Indirectness	Imprecision	Other considerations	GMT	Control	Relative (95% CI)	Absolute		
**GMT(Number of doses) (Better indicated by lower values)**												
25	randomised trials	no serious risk of bias	no serious inconsistency	no serious indirectness	no serious imprecision	strong association dose response gradient	2312	1072	-	SMD 0.92 higher (0.64 to 1.2 higher)	ÅÅÅÅ HIGH	CRITICAL
**GMT(Number of doses) - One dose (Better indicated by lower values)**												
7	randomised trials	no serious risk of bias	no serious inconsistency	no serious indirectness	no serious imprecision	none	729	304	-	SMD 0.84 higher (0.66 to 1.02 higher)	ÅÅÅÅ HIGH	IMPORTANT
**GMT(Number of doses) - Two dose (Better indicated by lower values)**												
14	randomised trials	no serious risk of bias	no serious inconsistency	no serious indirectness	no serious imprecision	none	1452	628	-	SMD 0.73 higher (0.56 to 0.9 higher)	ÅÅÅÅ HIGH	IMPORTANT
**GMT(Number of doses) - Three dose (Better indicated by lower values)**												
4	randomised trials	no serious risk of bias	no serious inconsistency	no serious indirectness	no serious imprecision	none	131	140	-	SMD 2.95 higher (0.65 lower to 6.55 higher)	ÅÅÅÅ HIGH	IMPORTANT
**GMT(Antibody type) (Better indicated by lower values)**												
25	randomised trials	no serious risk of bias	no serious inconsistency	no serious indirectness	no serious imprecision	none	2312	1072	-	SMD 0.92 higher (0.64 to 1.2 higher)	ÅÅÅÅ HIGH	IMPORTANT
**GMT(Antibody type) - Neutralizing antibodies (Better indicated by lower values)**												
13	randomised trials	no serious risk of bias	no serious inconsistency	no serious indirectness	no serious imprecision	none	1363	526	-	SMD 0.82 higher (0.64 to 1.01 higher)	ÅÅÅÅ HIGH	IMPORTANT
**GMT(Antibody type) - Anti-S (Better indicated by lower values)**												
7	randomised trials	no serious risk of bias	no serious inconsistency	no serious indirectness	no serious imprecision	none	501	378	-	SMD 1.11 higher (0.08 to 2.15 higher)	ÅÅÅÅ HIGH	IMPORTANT
**GMT(Antibody type) - Anti-RBD (Better indicated by lower values)**												
5	randomised trials	no serious risk of bias	no serious inconsistency	no serious indirectness	no serious imprecision	none	448	168	-	SMD 0.88 higher (0.44 to 1.31 higher)	ÅÅÅÅ HIGH	IMPORTANT
**GMT(Vaccine type) (Better indicated by lower values)**												
25	randomised trials	no serious risk of bias	no serious inconsistency	no serious indirectness	no serious imprecision	none	2312	1072	-	SMD 0.92 higher (0.64 to 1.2 higher)	ÅÅÅÅ HIGH	IMPORTANT
**GMT(Vaccine type) - Inactivated vaccine (Better indicated by lower values)**												
3	randomised trials	no serious risk of bias	no serious inconsistency	no serious indirectness	no serious imprecision	none	415	122	-	SMD 0.76 higher (0.23 to 1.29 higher)	ÅÅÅÅ HIGH	IMPORTANT
**GMT(Vaccine type) - Subunit vaccine (Better indicated by lower values)**												

6	randomised trials	no serious risk of bias	no serious inconsistency	no serious indirectness	no serious imprecision	none	991	292	-	SMD 0.91 higher (0.77 to 1.04 higher)	ÅÅÅÅ HIGH	IMPORTANT
**GMT(Vaccine type) - Nucleic acid vaccine (Better indicated by lower values)**												
7	randomised trials	no serious risk of bias	no serious inconsistency	no serious indirectness	no serious imprecision	none	224	233	-	SMD 1.57 higher (0.04 to 3.11 higher)	ÅÅÅÅ HIGH	IMPORTANT
**GMT(Vaccine type) - Viral vector vaccines (Better indicated by lower values)**												
9	randomised trials	no serious risk of bias	no serious inconsistency	no serious indirectness	no serious imprecision	none	682	425	-	SMD 0.67 higher (0.46 to 0.88 higher)	ÅÅÅÅ HIGH	IMPORTANT
**Seroconversion**												
10	randomised trials	no serious risk of bias	serious	no serious indirectness	no serious imprecision	strong association	-	-	-	OR 24.42 (19.29 to 30.92)-	ÅÅÅO MODERATE	CRITICAL
								0%				

Quality assessment							No of patients		Effect		Quality	Importance
**No of studies**	**Design**	**Risk of bias**	**Inconsistency**	**Indirectness**	**Imprecision**	**Other considerations**	**Vaccine safety**	**Control**	**Relative (95% CI)**	**Absolute**		
**Vaccine safety**												
11	randomised trials	no serious risk of bias	no serious inconsistency	no serious indirectness	no serious imprecision	strong association	5238/14297 (36.6%)	1299/6290 (20.7%)	OR 2.57 (1.83 to 3.62)	194 more per 1000 (from 116 more to 279 more)	ÅÅÅÅ HIGH	CRITICAL
								14.3%		157 more per 1000 (from 91 more to 234 more)		
**Vaccine safety - Total AEs**												
7	randomised trials	no serious risk of bias	no serious inconsistency	no serious indirectness	no serious imprecision	none	275/1126 (24.4%)	77/318 (24.2%)	OR 3.39 (1.01 to 11.4)	278 more per 1000 (from 2 more to 542 more)	ÅÅÅÅ HIGH	IMPORTANT
								16.7%		238 more per 1000 (from 1 more to 529 more)		
**Vaccine safety - Solicited local AEs**												
9	randomised trials	no serious risk of bias	no serious inconsistency	no serious indirectness	no serious imprecision	none	2440/6147 (39.7%)	418/2819 (14.8%)	OR 6.45 (2.78 to 14.97)	381 more per 1000 (from 178 more to 574 more)	ÅÅÅÅ HIGH	IMPORTANT
								6.4%		242 more per 1000 (from 96 more to 442 more)		
**Vaccine safety - Solicited systemic AEs**												

9	randomised trials	no serious risk of bias	no serious inconsistency	no serious indirectness	no serious imprecision	none	2415/6147 (39.3%)	760/2818 (27%)	OR 1.9 (1.24 to 2.92)	143 more per 1000 (from 44 more to 249 more)	ÅÅÅÅ HIGH	IMPORTANT
								16.7%		109 more per 1000 (from 32 more to 202 more)		
**Vaccine safety - Geriatric complications**												
2	randomised trials	no serious risk of bias	no serious inconsistency	no serious indirectness	no serious imprecision	none	108/877 (12.3%)	44/335 (13.1%)	OR 1.2 (0.82 to 1.76)	22 more per 1000 (from 21 fewer to 79 more)	ÅÅÅÅ HIGH	IMPORTANT
								9.5%		17 more per 1000 (from 16 fewer to 61 more)		
**Solicited local AE**												
11	randomised trials	no serious risk of bias	no serious inconsistency	no serious indirectness	no serious imprecision	none	1922/14127 (13.6%)	269/6168 (4.4%)	OR 3.82 (2.19 to 6.65)	105 more per 1000 (from 47 more to 189 more)	ÅÅÅÅ HIGH	IMPORTANT
								0%		-		
**Solicited local AE - Pain**												
11	randomised trials	no serious risk of bias	no serious inconsistency	no serious indirectness	no serious imprecision	none	1694/6579 (25.7%)	259/2918 (8.9%)	OR 5.04 (2.15 to 11.83)	241 more per 1000 (from 84 more to 447 more)	ÅÅÅÅ HIGH	IMPORTANT
								5.6%		174 more per 1000 (from 57 more to 356 more)		
**Solicited local AE - Swilling**												
8	randomised trials	no serious risk of bias	no serious inconsistency	no serious indirectness	no serious imprecision	none	90/1329 (6.8%)	4/412 (1%)	OR 3.31 (0.89 to 12.28)	22 more per 1000 (from 1 fewer to 98 more)	ÅÅÅÅ HIGH	IMPORTANT
								0%		-		
**Solicited local AE - Redness**												
10	randomised trials	no serious risk of bias	no serious inconsistency	no serious indirectness	no serious imprecision	none	138/6219 (2.2%)	6/2838 (0.2%)	OR 3.13 (0.9 to 10.94)	4 more per 1000 (from 0 fewer to 21 more)	ÅÅÅÅ HIGH	IMPORTANT
								0%		-		
**Solicited systemic AE**												
12	randomised trials	no serious risk of bias	no serious inconsistency	no serious indirectness	no serious imprecision	none	2678/19545 (13.7%)	723/8639 (8.4%)	OR 1.91 (1.75 to 2.09)	65 more per 1000 (from 54 more to 77 more)	ÅÅÅÅ HIGH	IMPORTANT
								1.3%		12 more per 1000 (from 10 more to 14 more)		
**Solicited systemic AE - Fever**												
12	randomised trials	no serious risk of bias	no serious inconsistency	no serious indirectness	no serious imprecision	none	132/6862 (1.9%)	5/2998 (0.2%)	OR 5.38 (2.79 to 10.37)	7 more per 1000 (from 3 more to 15 more)	ÅÅÅÅ HIGH	IMPORTANT
								0%		-		

**Solicited systemic AE - Fatigue**												
10	randomised trials	no serious risk of bias	no serious inconsistency	no serious indirectness	no serious imprecision	none	1348/6327 (21.3%)	420/2833 (14.8%)	OR 1.65 (1.46 to 1.86)	75 more per 1000 (from 54 more to 96 more)	ÅÅÅÅ HIGH	IMPORTANT
								11.7%		62 more per 1000 (from 45 more to 81 more)		
**Solicited systemic AE - Headache**												
10	randomised trials	no serious risk of bias	no serious inconsistency	no serious indirectness	no serious imprecision	none	1198/6356 (18.8%)	298/2808 (10.6%)	OR 2.12 (1.85 to 2.44)	95 more per 1000 (from 74 more to 118 more)	ÅÅÅÅ HIGH	IMPORTANT
								5.4%		54 more per 1000 (from 42 more to 68 more)		

Appendix 9. The shape of the funnel plot of vaccine effectiveness (including number of doses, vaccine type), the shape of the funnel plot of GMT (including antibody type, vaccine type), and the shape of the funnel plot of solicited local adverse event and solicited systemic adverse event

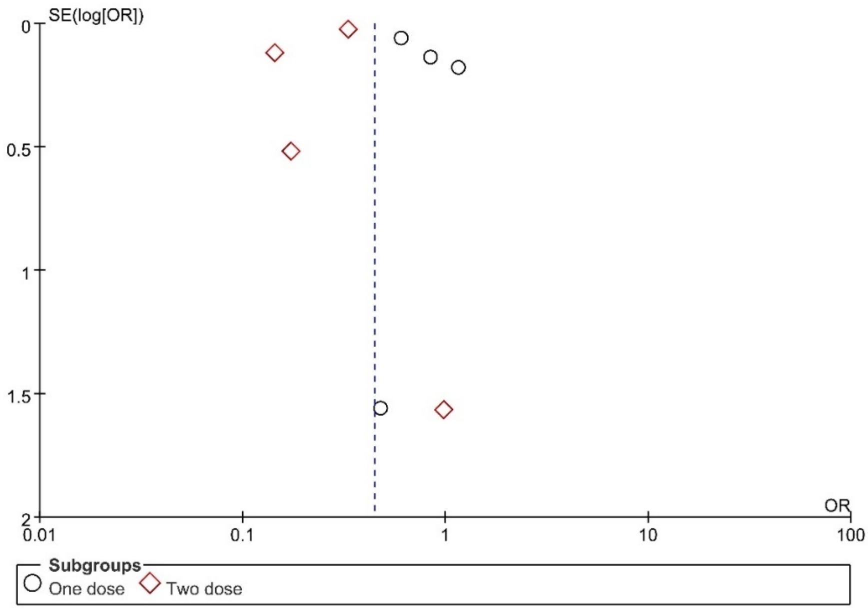

Figure 9-1 The shape of the funnel plot of vaccine effectiveness (number of doses)

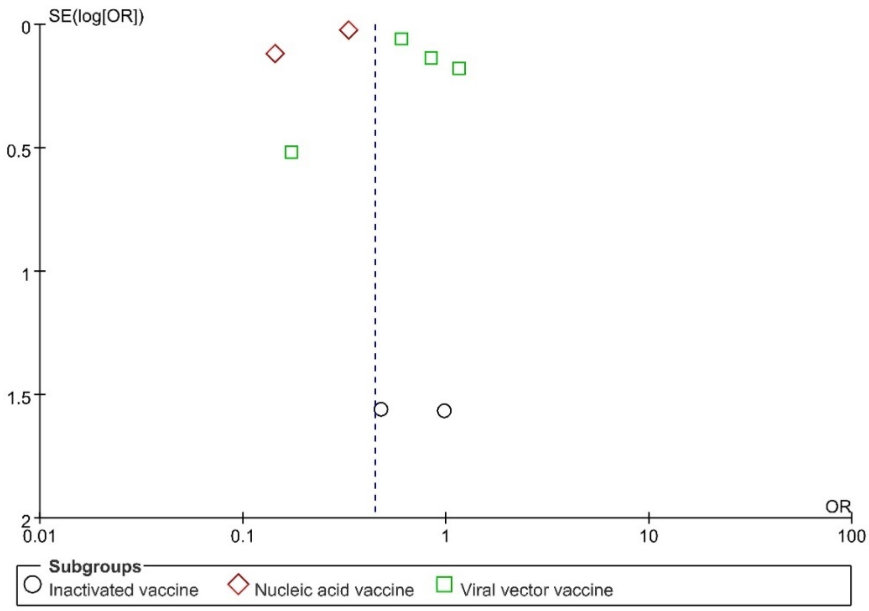

Figure 9-2 The shape of the funnel plot of vaccine effectiveness (vaccine type)

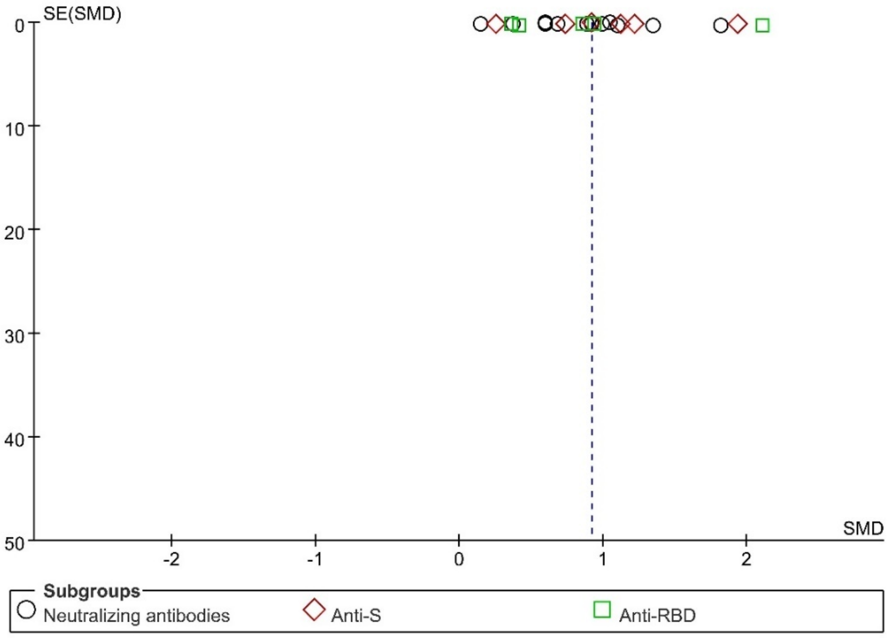

Figure 9-3 The shape of the funnel plot of GMT (antibody type)

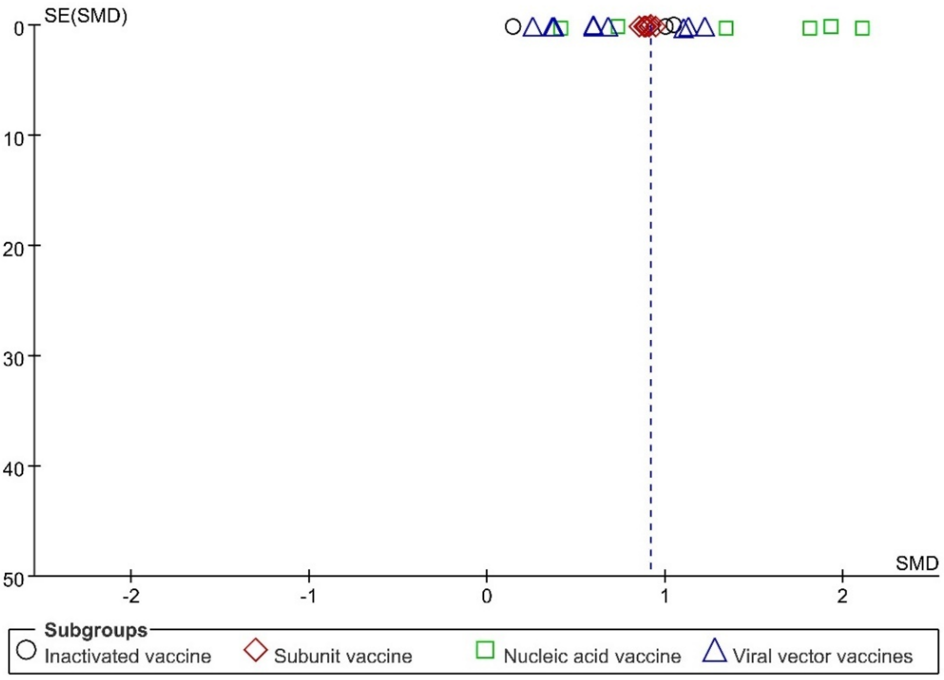

Figure 9-4 The shape of the funnel plot of GMT (vaccine type)

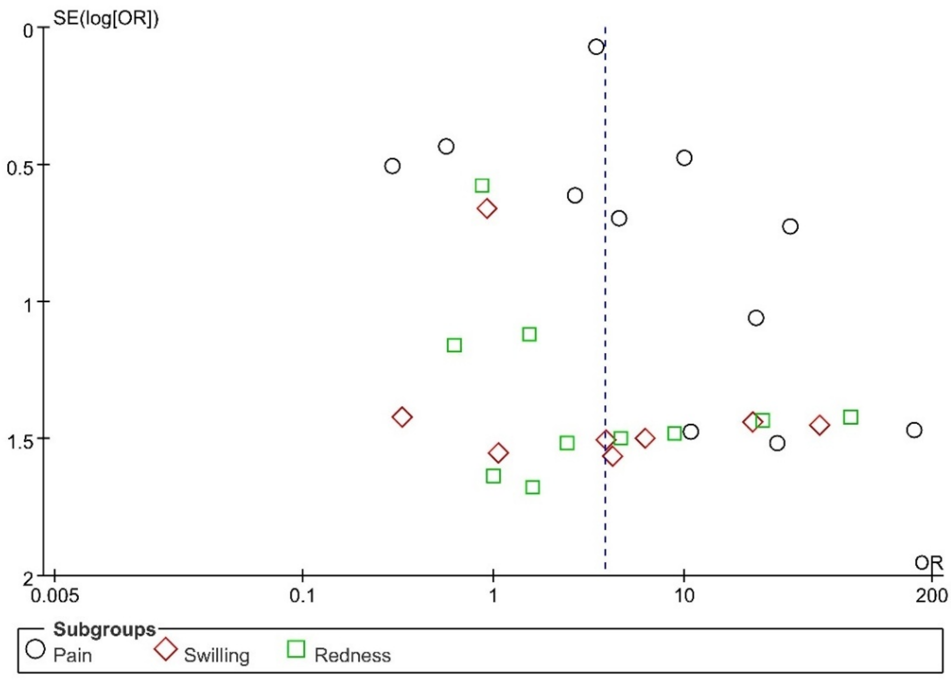

Figure 9-5 The shape of the funnel plot of solicited local adverse event

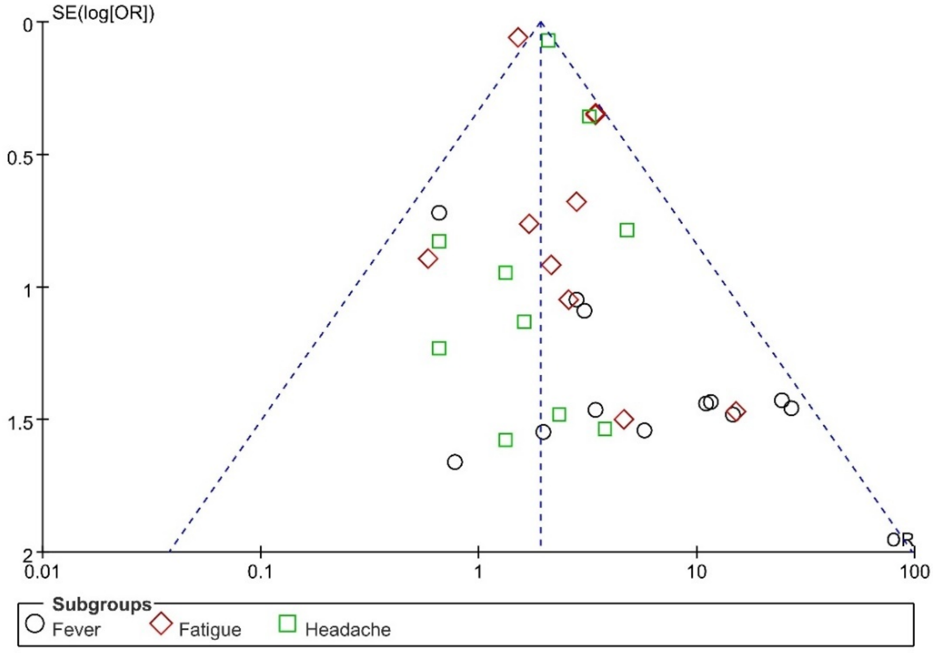

Figure 9-6 The shape of the funnel plot of solicited systemic adverse event

Appendix 10. Egger test

Outcomes	t	df	P value
**Vaccine effectiveness**	0.61	6	0.5620
Infection after vaccination	0.35	1	0.7852
**Vaccine effectiveness (Number of doses)**	0.61	6	0.5620
One dose	1.06	2	0.3996
Two doses	-0.87	2	0.4772
**Vaccine effectiveness (Vaccine type)**	0.61	6	0.5620
Viral vector vaccine	0.20	2	0.8630
**GMT**	3.63	23	0.0014
Two doses	0.45	12	0.6590
Three doses	4.27	2	0.0507
**GMT (Antibody type)**	3.63	23	0.0014
Neutralizing antibodies	1.12	11	0.2883
Anti-S	3.39	5	0.0194
Anti-RBD	0.71	3	0.5274
**GMT (Vaccine type)**	3.63	23	0.0014
Nucleic acid vaccine	4.44	5	0.0067
Viral vector vaccine	1.23	7	0.2572
**Vaccine safety**	0.69	25	0.4960
Total AEs	0.97	5	0.3761
Solicited local AEs	0.90	7	0.3982
Solicited systemic AEs	0.31	7	0.7637
**Solicited local AE (Subgroup)**	0.32	27	0.7509
Pain	0.30	9	0.7696
Swilling	1.52	6	0.1798
Redness	1.99	8	0.0819

t, student's t test; df, degree of freedom; p, p-value.

Appendix 11. The shape of the forest plot of vaccine effectiveness (including number of doses, vaccine type), the shape of the forest plot of GMT (including antibody type, vaccine type), the shape of the forest plot of solicited local adverse event and solicited systemic adverse event

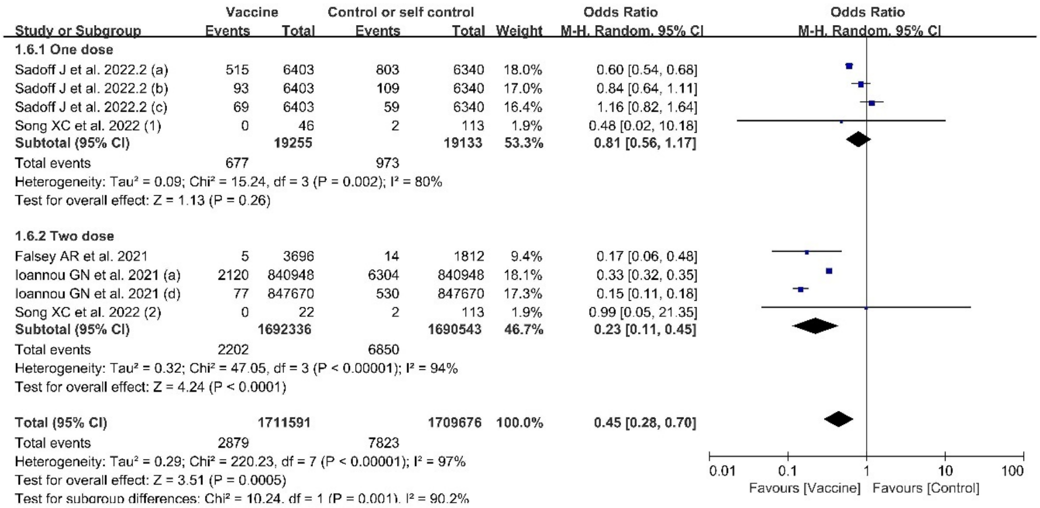

Figure 11-1 The shape of the forest plot of vaccine effectiveness (number of doses). (a) infection after vaccination; (b) hospitalized after vaccination; (c) ICU after vaccination; (d) death after vaccination; (1) one dose; (2) two doses.

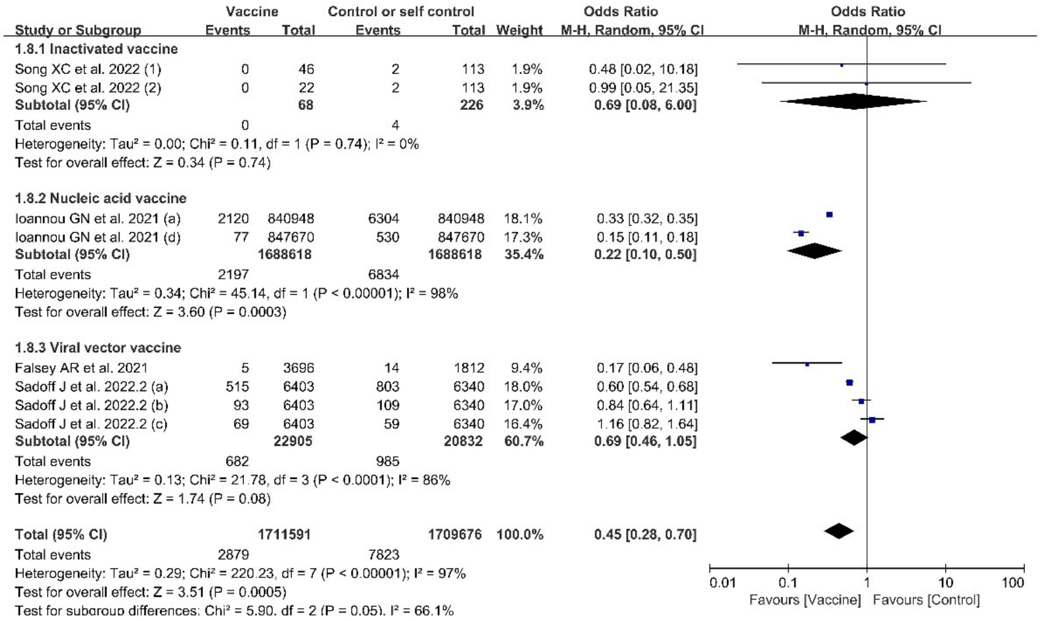

Figure 11-2 The shape of the forest plot of vaccine effectiveness (vaccine type). (a) infection after vaccination; (b) hospitalized after vaccination; (c) ICU after vaccination; (d) death after vaccination; (1) one dose; (2) two doses.

Study or Subgroup	Vaccine Mean	Vaccine SD	Vaccine Total	Control or self control Mean	Control or self control SD	Control or self control Total	Weight	Std. Mean Difference IV, Random, 95% CI
**2.3.1 Neutralizing antibodies**								
Formica N et al. 2021 (N)	1,007.35	1,344.08	52	10	0.0001	25	4.1%	0.89 [0.39, 1.39]
Kundro MA et al. 2022 (I)	1.197	1.141	27	0.9026	2.534	27	4.0%	0.15 [-0.39, 0.68]
Kundro MA et al. 2022 (V)	1.947	2.751	58	0.9026	2.788	58	4.4%	0.37 [0.01, 0.74]
Ramasamy MN et al. 2020 (N1)	44	91.958	47	5	0.0001	47	4.3%	0.59 [0.18, 1.01]
Ramasamy MN et al. 2020 (N2)	146	297.31	43	5	0.0001	47	4.3%	0.68 [0.25, 1.11]
Sadoff J et al. 2022	503.2	605.31	24	21.834	0.0001	24	3.9%	1.11 [0.50, 1.72]
Sadoff J et al. 2022.6	424.236	696.62	322	50.873	0.0001	81	4.5%	0.60 [0.35, 0.85]
Sáez-Llorens X et al. 2022 (N2)	61.946	55.733	21	7.863	2.281	21	3.7%	1.35 [0.67, 2.02]
Sáez-Llorens X et al. 2022 (N3)	89.43	62.359	21	7.863	2.281	21	3.6%	1.81 [1.08, 2.54]
Shu YJ et al. 2021 (N1)	21.4	21.238	120	5	0.0001	40	4.4%	0.89 [0.51, 1.26]
Shu YJ et al. 2021 (N2)	108.65	121.38	240	5.4	1.72	40	4.4%	0.91 [0.57, 1.26]
Wu Z et al. 2021 (N1)	8	7.255	48	2	0.0001	24	4.1%	1.00 [0.48, 1.52]
Wu Z et al. 2021 (N2)	41.38	41.142	340	2.1	0.146	71	4.5%	1.05 [0.78, 1.31]
**Subtotal (95% CI)**			**1363**			**526**	**54.1%**	**0.82 [0.64, 1.01]**
Heterogeneity: Tau² = 0.06; Chi² = 30.21, df = 12 (P = 0.003); I² = 60%								
Test for overall effect: Z = 8.73 (P < 0.00001)								
**2.3.2 Anti-S**								
Alidjinou EK et al. 2022 (S2)	529	1,006.2	51	0	4.6044	51	4.3%	0.74 [0.34, 1.14]
Alidjinou EK et al. 2022 (S3)	2,080	0.0001	44	0	4.6044	51	0.0%	611.11 [522.36, 699.87]
Formica N et al. 2021 (S)	30,406.61	40,146.5	219	127.8	106.61	107	4.6%	0.92 [0.68, 1.16]
Lanini S et al. 2022 (S)	5,297.048	28,271.707	46	42.029	0.0001	46	4.3%	0.26 [-0.15, 0.67]
Poh XY et al. 2022	21,121.07	15,139.89	45	452.53	367.91	47	4.1%	1.94 [1.44, 2.43]
Ramasamy MN et al. 2020 (S1)	84.91	95.086	48	2.07	1.6186	48	4.2%	1.22 [0.78, 1.66]
Ramasamy MN et al. 2020 (S2)	471.51	516.79	48	2.11	1.7392	28	4.1%	1.13 [0.63, 1.63]
**Subtotal (95% CI)**			**501**			**378**	**25.6%**	**1.11 [0.08, 2.15]**
Heterogeneity: Tau² = 1.64; Chi² = 210.49, df = 6 (P < 0.00001); I² = 97%								
Test for overall effect: Z = 2.10 (P = 0.04)								
**2.3.3 Anti-RBD**								
Lanini S et al. 2022 (R)	3,501.138	13,301.91	46	42.029	0.0001	46	4.3%	0.36 [-0.05, 0.78]
Sáez-Llorens X et al. 2022 (R2)	972.727	2,948.6	21	84.464	31.118	21	3.9%	0.42 [-0.19, 1.03]
Sáez-Llorens X et al. 2022 (R3)	5,446.43	3,530.7	21	84.464	31.118	21	3.5%	2.11 [1.34, 2.88]
Shu YJ et al. 2021 (R1)	654.2	871.05	120	5.6	0.77	40	4.4%	0.85 [0.48, 1.22]
Shu YJ et al. 2021 (R2)	2,665.6	3,031.6	240	5.71	1.4	40	4.4%	0.94 [0.60, 1.29]
**Subtotal (95% CI)**			**448**			**168**	**20.4%**	**0.88 [0.44, 1.31]**
Heterogeneity: Tau² = 0.18; Chi² = 17.65, df = 4 (P = 0.001); I² = 77%								
Test for overall effect: Z = 3.94 (P < 0.0001)								
**Total (95% CI)**			**2312**			**1072**	**100.0%**	**0.92 [0.64, 1.20]**
Heterogeneity: Tau² = 0.43; Chi² = 261.27, df = 24 (P < 0.00001); I² = 91%								
Test for overall effect: Z = 6.41 (P < 0.00001)								
Test for subgroup differences: Chi² = 0.32, df = 2 (P = 0.85), I² = 0%								

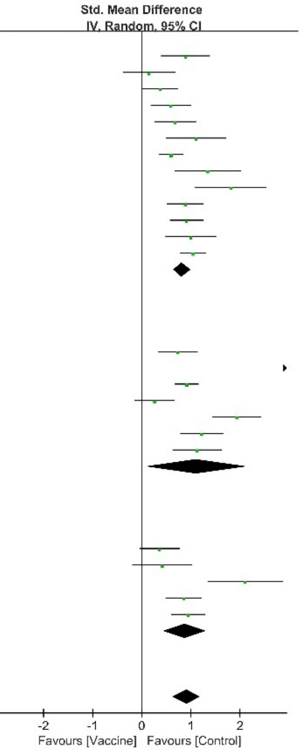

Figure 11-3 The shape of the forest plot of GMT (antibody type). (N) neutralizing antibodies; (S) anti-S antibodies; (R) anti-RBD antibodies; (1) one dose; (2) two doses; (3) three doses; (I) inactivated vaccine; (V) viral vector vaccine.

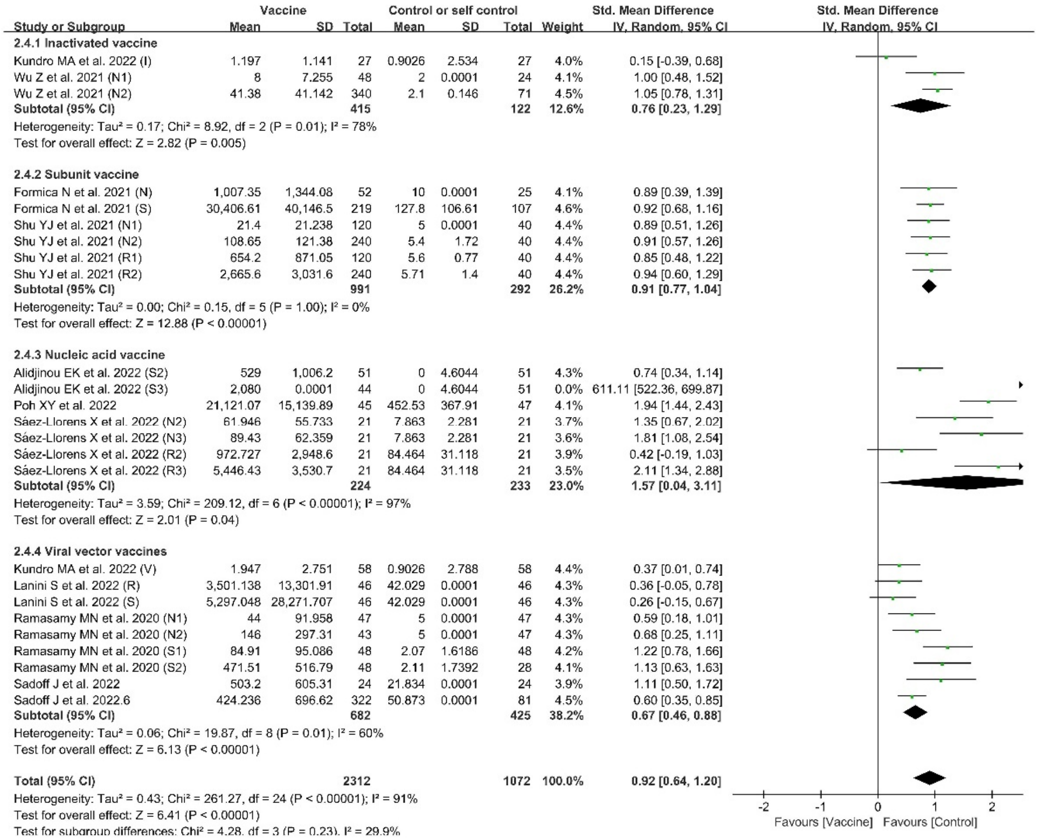

Figure 11-4 The shape of the forest plot of GMT (vaccine type). (N) neutralizing antibodies; (S) anti-S antibodies; (R) anti-RBD antibodies; (1) one dose; (2) two doses; (3) three doses.

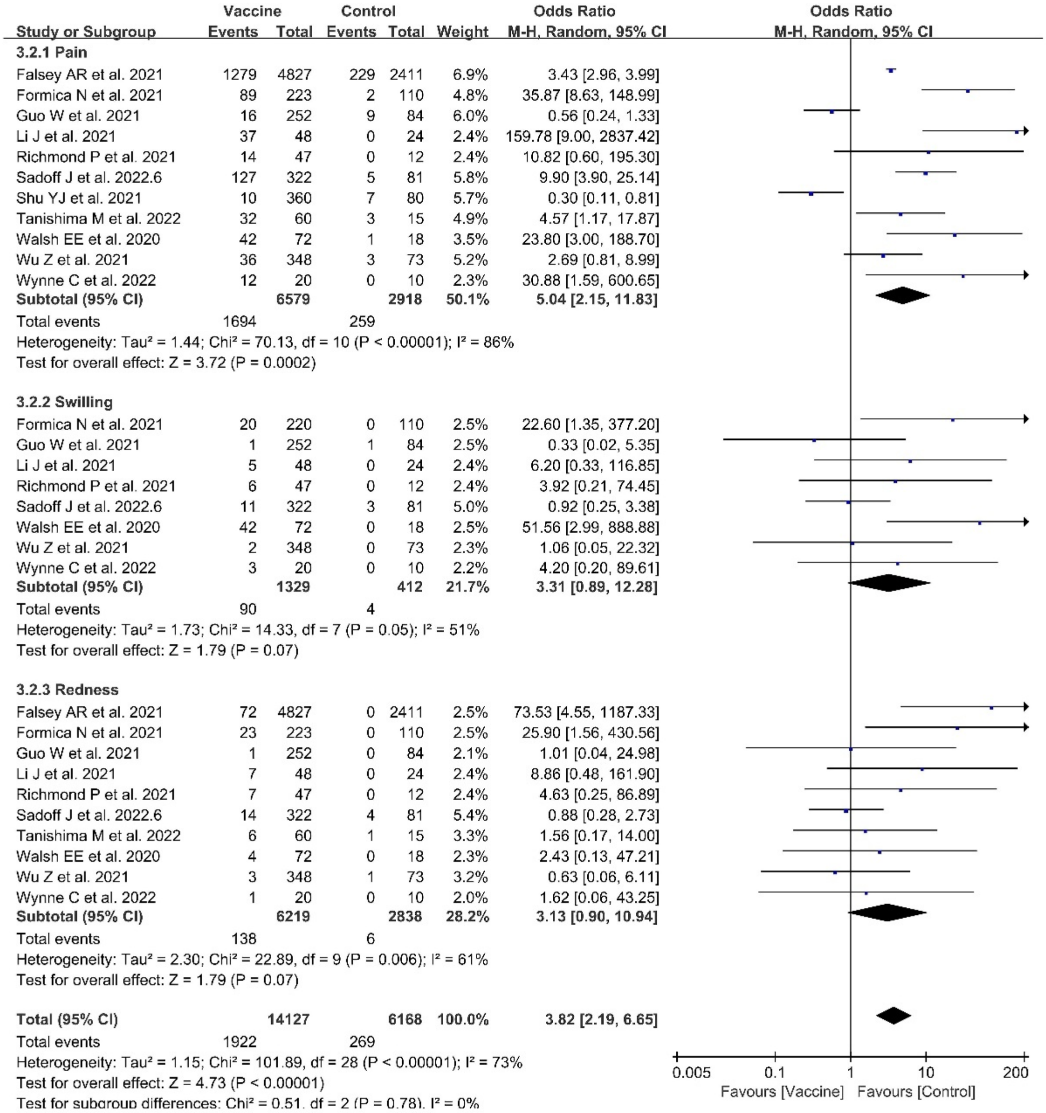

Study or Subgroup	Vaccine Events	Vaccine Total	Control Events	Control Total	Weight	Odds Ratio M-H, Random, 95% CI
**3.2.1 Pain**						
Falsey AR et al. 2021	1279	4827	229	2411	6.9%	3.43 [2.96, 3.99]
Formica N et al. 2021	89	223	2	110	4.8%	35.87 [8.63, 148.99]
Guo W et al. 2021	16	252	9	84	6.0%	0.56 [0.24, 1.33]
Li J et al. 2021	37	48	0	24	2.4%	159.78 [9.00, 2837.42]
Richmond P et al. 2021	14	47	0	12	2.4%	10.82 [0.60, 195.30]
Sadoff J et al. 2022.6	127	322	5	81	5.8%	9.90 [3.90, 25.14]
Shu YJ et al. 2021	10	360	7	80	5.7%	0.30 [0.11, 0.81]
Tanishima M et al. 2022	32	60	3	15	4.9%	4.57 [1.17, 17.87]
Walsh EE et al. 2020	42	72	1	18	3.5%	23.80 [3.00, 188.70]
Wu Z et al. 2021	36	348	3	73	5.2%	2.69 [0.81, 8.99]
Wynne C et al. 2022	12	20	0	10	2.3%	30.88 [1.59, 600.65]
**Subtotal (95% CI)**		**6579**		**2918**	**50.1%**	**5.04 [2.15, 11.83]**
Total events	1694		259			
Heterogeneity: Tau² = 1.44; Chi² = 70.13, df = 10 (P < 0.00001); I² = 86%						
Test for overall effect: Z = 3.72 (P = 0.0002)						
**3.2.2 Swilling**						
Formica N et al. 2021	20	220	0	110	2.5%	22.60 [1.35, 377.20]
Guo W et al. 2021	1	252	1	84	2.5%	0.33 [0.02, 5.35]
Li J et al. 2021	5	48	0	24	2.4%	6.20 [0.33, 116.85]
Richmond P et al. 2021	6	47	0	12	2.4%	3.92 [0.21, 74.45]
Sadoff J et al. 2022.6	11	322	3	81	5.0%	0.92 [0.25, 3.38]
Walsh EE et al. 2020	42	72	0	18	2.5%	51.56 [2.99, 888.88]
Wu Z et al. 2021	2	348	0	73	2.3%	1.06 [0.05, 22.32]
Wynne C et al. 2022	3	20	0	10	2.2%	4.20 [0.20, 89.61]
**Subtotal (95% CI)**		**1329**		**412**	**21.7%**	**3.31 [0.89, 12.28]**
Total events	90		4			
Heterogeneity: Tau² = 1.73; Chi² = 14.33, df = 7 (P = 0.05); I² = 51%						
Test for overall effect: Z = 1.79 (P = 0.07)						
**3.2.3 Redness**						
Falsey AR et al. 2021	72	4827	0	2411	2.5%	73.53 [4.55, 1187.33]
Formica N et al. 2021	23	223	0	110	2.5%	25.90 [1.56, 430.56]
Guo W et al. 2021	1	252	0	84	2.1%	1.01 [0.04, 24.98]
Li J et al. 2021	7	48	0	24	2.4%	8.86 [0.48, 161.90]
Richmond P et al. 2021	7	47	0	12	2.4%	4.63 [0.25, 86.89]
Sadoff J et al. 2022.6	14	322	4	81	5.4%	0.88 [0.28, 2.73]
Tanishima M et al. 2022	6	60	1	15	3.3%	1.56 [0.17, 14.00]
Walsh EE et al. 2020	4	72	0	18	2.3%	2.43 [0.13, 47.21]
Wu Z et al. 2021	3	348	1	73	3.2%	0.63 [0.06, 6.11]
Wynne C et al. 2022	1	20	0	10	2.0%	1.62 [0.06, 43.25]
**Subtotal (95% CI)**		**6219**		**2838**	**28.2%**	**3.13 [0.90, 10.94]**
Total events	138		6			
Heterogeneity: Tau² = 2.30; Chi² = 22.89, df = 9 (P = 0.006); I² = 61%						
Test for overall effect: Z = 1.79 (P = 0.07)						
**Total (95% CI)**		**14127**		**6168**	**100.0%**	**3.82 [2.19, 6.65]**
Total events	1922		269			
Heterogeneity: Tau² = 1.15; Chi² = 101.89, df = 28 (P < 0.00001); I² = 73%						
Test for overall effect: Z = 4.73 (P < 0.00001)						
Test for subgroup differences: Chi² = 0.51, df = 2 (P = 0.78), I² = 0%						

Figure 11-5 The shape of the forest plot of solicited local adverse event

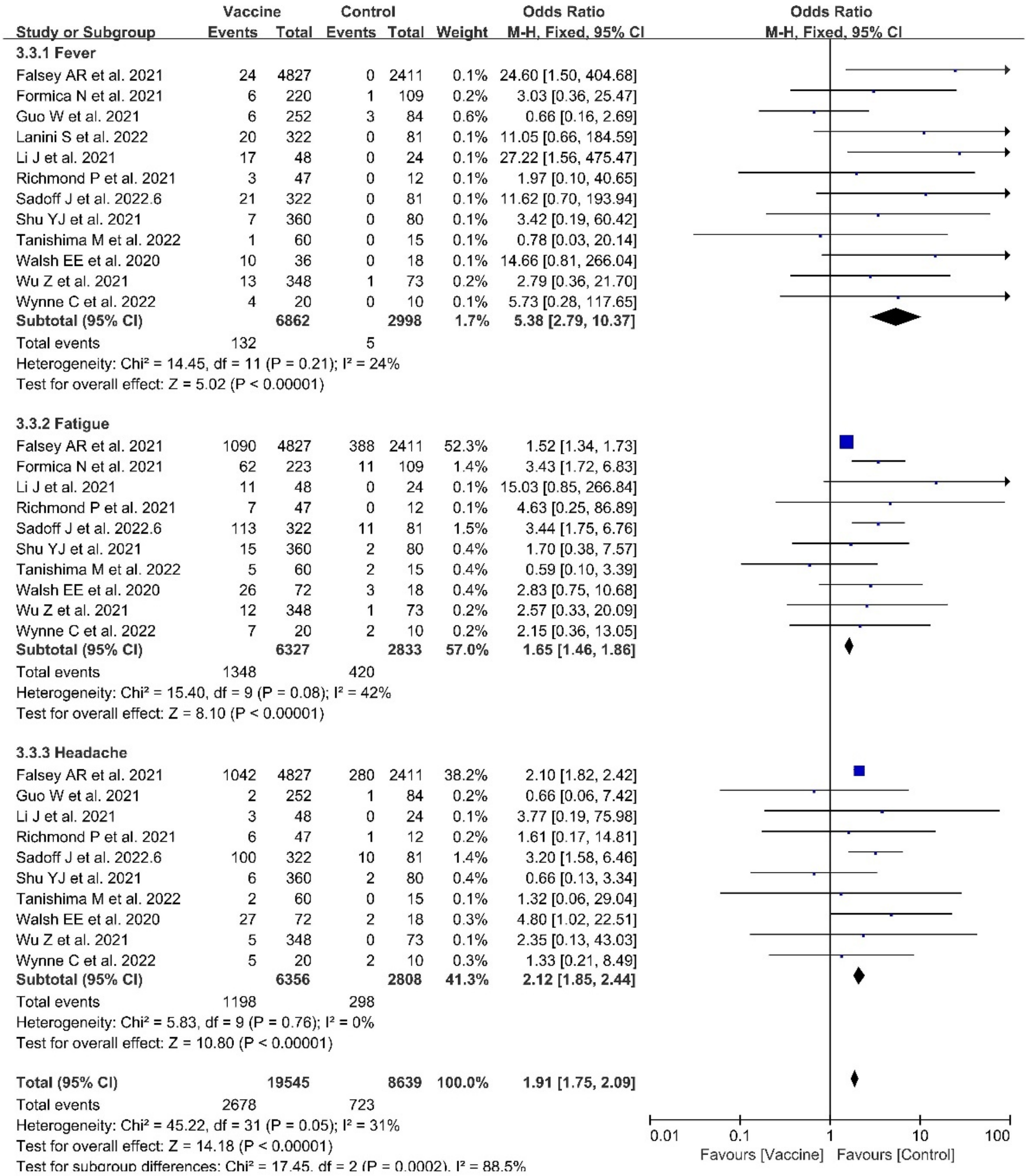

Figure 11-6 The shape of the forest plot of solicited systemic adverse event
